# Chemokine receptor trafficking coordinates neutrophil clustering and dispersal at wounds in zebrafish

**DOI:** 10.1038/s41467-019-13107-3

**Published:** 2019-11-14

**Authors:** Caroline Coombs, Antonios Georgantzoglou, Hazel A. Walker, Julian Patt, Nicole Merten, Hugo Poplimont, Elisabeth M. Busch-Nentwich, Sarah Williams, Christina Kotsi, Evi Kostenis, Milka Sarris

**Affiliations:** 1 Department of Physiology, Development and Neuroscience, University of Cambridge, Downing Site, Cambridge, CB2 3DY UK; 2 Institute for Pharmaceutical Biology, University of Bonn, Nussallee 6, Bonn, 53115 Germany; 3 Wellcome Sanger Institute, Wellcome Genome Campus, Hinxton, CB10 1SA UK; 4 Department of Medicine, University of Cambridge, Cambridge, CB2 0QQ UK

**Keywords:** Amoeboid migration, Chemotaxis, Imaging the immune system, Neutrophils

## Abstract

Immune cells congregate at specific loci to fight infections during inflammatory responses, a process that must be transient and self-resolving. Cell dispersal promotes resolution, but it remains unclear how transition from clustering to dispersal is regulated. Here we show, using quantitative live imaging in zebrafish, that differential ligand-induced trafficking of chemokine receptors such as Cxcr1 and Cxcr2 orchestrates the state of neutrophil congregation at sites of tissue damage. Through receptor mutagenesis and biosensors, we show that Cxcr1 promotes clustering at wound sites, but is promptly desensitized and internalized, which prevents excess congregation. By contrast, Cxcr2 promotes bidirectional motility and is sustained at the plasma membrane. Persistent plasma membrane residence of Cxcr2 prolongs downstream signaling and is required for sustained exploratory motion conducive to dispersal. Thus, differential trafficking of two chemokine receptors allows coordination of antagonistic cell behaviors, promoting a self-resolving migratory response.

## Abstract (web summary)

Inflammatory responses must be induced and resolved timely to serve protection from pathogens without inducing excessive tissue damage. Here the authors use live imaging in zebrafish to show that the intracellular trafficking of two chemokine receptors, Cxcr1 and Cxcr2, is differentially regulated on activated neutrophils to control their clustering and dispersal, respectively.

## Introduction

Cell migration is required in development and immune responses, and is the hallmark of metastasis. Cells are directed towards target sites by chemoattractants, often in the form of secreted molecules that are recognized by G-protein-coupled receptors (GPCRs). GPCR signaling activates effectors of cytoskeletal dynamics that promote directed migration (chemotaxis)^1,2^. The final distribution of cells in a tissue depends on their behavior post arrival at the target. Upon reaching a source of attractant, cells may stop and cluster or may disperse and leave the site. There is a trade-off between the two behaviors. Clustering allows close inspection of an area and maximal response to local signals, be it growth factors, nutrients, activation cues, or microbes. Yet, this occurs at the expense of global space exploration and the encounter of alternative signals nearby. It remains unclear how cell dispersal and clustering are balanced to generate specific large-scale physiological or pathological outcomes.

The inflammatory response represents a prominent example where cell dispersal and clustering must be tightly controlled. Neutrophils are the first cells to be recruited to sites of damage or infection where they promote innate immunity through phagocytosis, release of antimicrobial products and production of signals that recruit or activate other immune cells^3^. Neutrophils are also recruited to tumors and can modulate the inflammatory niche and tumor evolution^4^. Excess neutrophil presence is often detrimental, as it may perpetuate inflammation and cause collateral tissue damage through neutrophil-released enzymes or radicals^3^. Thus, altogether, there is strong biomedical interest in understanding how neutrophil migration is controlled and resolved.

Neutrophils reach sites of infection or damage after extravasation and chemotaxis to inflammatory chemoattractants, such as leukotrienes, pathogen-associated molecular patterns, damage-associated molecular patterns, and chemokines, such as CXCL8 (interleukin-8)^3,5^. A long-held view has been that neutrophil recruitment is resolved through apoptosis at sites of inflammation. However, increasing evidence suggests neutrophils can also depart from sites of inflammation revealing an additional step in resolution^6–9^. This so-called reverse migration is distinguished into two stages: reverse interstitial migration (rIM), i.e., extra-vascular movement away from the inflammatory focus, and reverse transmigration (rTEM), i.e., abluminal-to-luminal transmigration into the blood stream^9^. The mechanism of rIM remains elusive but cell tracking analyses indicate that random dispersal rather than repulsion plays a role^9,10^. Unexpectedly, some chemokine receptors stimulate random interstitial motility (chemokinesis) and as such drive dispersal^11,12^. This raises the question why a subset of chemoattractant receptors can promote clustering while others promote dispersal.

Candidate mechanisms for functional diversification of chemoattractant receptors lie in the early receptor signaling events after ligand binding. Ligand binding triggers GPCR conformational changes that induce association of trimeric αβγ G proteins, which are classified according to the variable α-subunit (Gs, Gi/o, Gq/11, or G12/13)^13^. GPCR activation triggers exchange of GDP for GTP on the α-subunit and dissociation of the trimer into a monomeric α-subunit and a dimeric βγ-subunit, which trigger various cytoskeletal effectors^1,13^. Subsequently, GPCRs are phosphorylated by GPCR kinases (GRKs). Phosphorylation sterically blocks re-association to G proteins, preventing further signaling, leading to the so-called desensitization^14–16^. Thereafter, GPCRs may be rapidly dephosphorylated and recycled at the plasma membrane or downregulated through degradation in lysosomes^14–16^. The role of receptor desensitization has been studied in WHIM syndrome neutrophils (Warts–Hypogammaglobulinemia–Immunodeficiency–Myelokathexis), B cells, and zebrafish primordial germ cells^17–20^. The impact of desensitization differs in these settings. Blocking receptor desensitization in WHIM syndrome neutrophils and in B cells inhibited passage of cells to tissues producing alternative chemoattractants^17,18^. In contrast, in primordial germ cells equivalent mutations led to cells skipping their target developmental site^20^. Furthermore, GRK inhibition can suppress or enhance gradient sensing in leukocyte chemotaxis under different settings in vitro^21–23^. The molecular basis for the different functional effects observed is unclear. Thus, it is difficult to infer how receptor trafficking might regulate neutrophil behavior within inflammatory sites.

Here we show that differential trafficking of two chemokine receptors coordinates neutrophil clustering and dispersal at sites of tissue damage. We link chemokine receptor trafficking with neutrophil behavior in zebrafish through genetic manipulations and live imaging. We demonstrate that the chemokine receptor Cxcr1 promotes neutrophil clustering but is rapidly desensitized and internalized in response to gradients of its ligand (Cxcl8a) at wounds. This is critical to allow transition to signaling through the chemokine receptor Cxcr2, which recognizes Cxcl8b and promotes bidirectional motion. Unlike Cxcr1, Cxcr2 is sustained at the plasma membrane of neutrophils. We show that this is required for sustained random motion and reverse migration that supports resolution of the response. These findings provide a foundation for identifying receptors that mediate neutrophil dispersal in mammals and for directing chemoattractant drug discovery towards specific leukocyte trafficking patterns.

## Results

### Rapid constitutive turnover of neutrophil Cxcr1 and Cxcr2

**Fig. 1:**
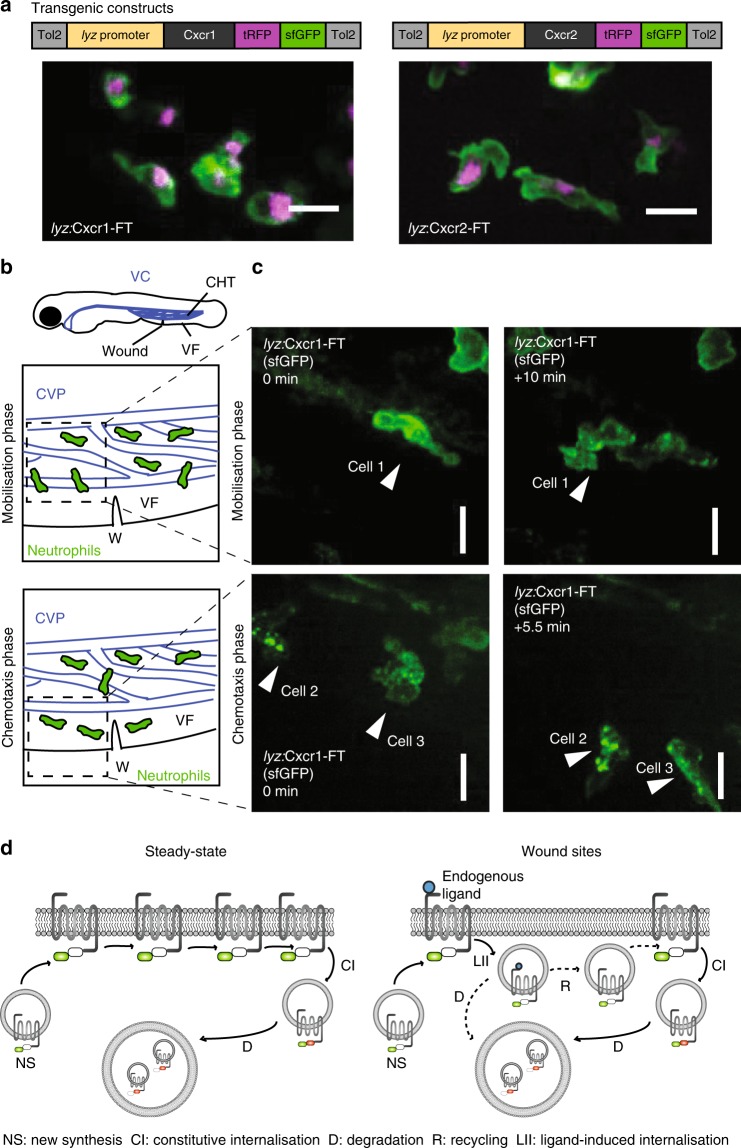
Live imaging of chemokine receptor trafficking in neutrophils. **a** Constructs used for neutrophil-specific transgenic expression of Cxcr1-FT (Fluorescent Timer) and Cxcr2-FT. Confocal projections of neutrophils in the head of a 3 days post fertilization (dpf) transgenic larva (Tg(*lyz*:Cxcr1-FT), top; Tg(*lyz*:Cxcr2-FT), bottom) showing tRFP (tagRFP; magenta) and sfGFP (green) channels. Scale bar = 20 µm. **b** Anatomical scheme of 3 dpf larva showing the location of the caudal hematopoietic tissue (CHT), the venus circulation (VC, blue), the ventral fin (VF), and the wound site. Below the larva are schemes depicting the area of the wound (W) with neutrophils getting mobilized from the CHT (top) or performing chemotaxis upon entering the ventral fin (bottom). The Caudal Vein plexus (CVP) of the CHT tissue is drawn in blue. Dashed square indicates area imaged in snapshots on the right. **c** Neutrophils in Tg(*lyz*:Cxcr1-FT) larvae (sfGFP is shown) upon mobilization from the CHT (top panels) or chemotaxis towards the wound (bottom panels). Arrows show the same cells over time. Time points on the right image are minutes elapsed after image on the left. Scale bar = 10 µm. **d** Schematic of Cxcr1/2-FT construct behavior in neutrophils. Newly synthesized receptors fluoresce in green due to short residence time at the plasma membrane. Older receptors fluoresce in red and green, and accumulate in intracellular compartments through constitutive turnover. Upon exposure to ligands at wounds, the receptor may internalize and subsequently be degraded or recycled

Neutrophil receptors for inflammatory chemoattractants^24,25^ show differential trafficking upon ligand binding, the functional significance of which in vivo is unclear. We hypothesized that receptor turnover or trafficking may diversify the contributions of chemoattractant receptors in the inflammatory response. To address this question, we sought to develop a model that allows imaging and manipulation of neutrophil receptor trafficking behavior in a physiological inflammatory setting in vivo. Zebrafish larvae are ideally amenable to imaging and genetic manipulation, and express homologs of the human CXCL8 receptors CXCR1 (Cxcr1) and CXCR2 (Cxcr2)^26^. We set out to visualize zebrafish Cxcr1 and Cxcr2 trafficking and turnover by generating fluorescent timer constructs (hereafter referred to as Cxcr1-FT and Cxcr2-FT)^27,28^. Cxcr1 and Cxcr2 were fused to tandems of superfolder green fluorescent protein (GFP) and tag red fluorescent protein (tagRFP) (Fig. 1a). Superfolder GFP (sfGFP) matures rapidly (<10 min) and allows monitoring of fast receptor dynamics, whereas tagRFP requires over 1.5 hours (h) to fluoresce^28^. sfGFP is quenched in acidic environments, whereas tagRFP remains stable. Thus, the combination of the two fluorophores allows monitoring of a broad range of receptor fates and can provide estimates of protein turnover time at the plasma membrane (newly synthesized receptors would fluoresce in green and progressively become red as they age).

To investigate the constitutive and ligand-induced dynamics of Cxcr1 and Cxcr2 in neutrophils, we generated transgenic fish expressing either receptor in neutrophils under the Lysozyme C promoter (Tg(*lyz*:Cxcr1-FT), Tg(*lyz*:Cxcr2-FT); Fig. 1a)^29,30^. Interestingly, neutrophils at steady state showed predominantly green fluorescence at the plasma membrane, whereas red receptors accumulated in intracellular, presumably more acidic, vesicular compartments (Fig. 1a and Supplementary Movie 1). This distribution was similar for Cxcr1-FT and Cxcr2-FT transgenics (Fig. 1a and Supplementary Movie 1). This indicated that both receptors have rapid constitutive turnover in these cells, and that the residence time at the plasma membrane is shorter than the maturation time of tagRFP (see below).

### Cxcr1 and Cxcr2 have distinct trafficking at wound sites

We then explored ligand-induced receptor trafficking based on the rapid dynamics of the sfGFP-labeled receptor molecules. We performed a wound in the ventral fin of a 3 day post fertilization (dpf) larva, nearby the caudal hematopoietic tissue (CHT), which is the first site of definitive hematopoiesis and a site of neutrophil production^31^ (Fig. 1b). We found that Cxcr1-FT first underwent internalization upon mobilization of the cells from the CHT (Fig. 1c and Supplementary Movies 2 and 3). Once in the ventral fin, neutrophils underwent chemotaxis concomitantly with further internalization of the Cxcr1-FT as they reached the wound (Fig. 1c and Supplementary Movies 2 and 3). In contrast with Cxcr1, Cxcr2 remained largely sustained at the cell membrane (Supplementary Movies 2 and 4). Thus, changes in the distribution of sfGFP could serve as a readout for receptor internalization in response to endogenous ligands at wounds (Fig. 1d).

**Fig. 2:**
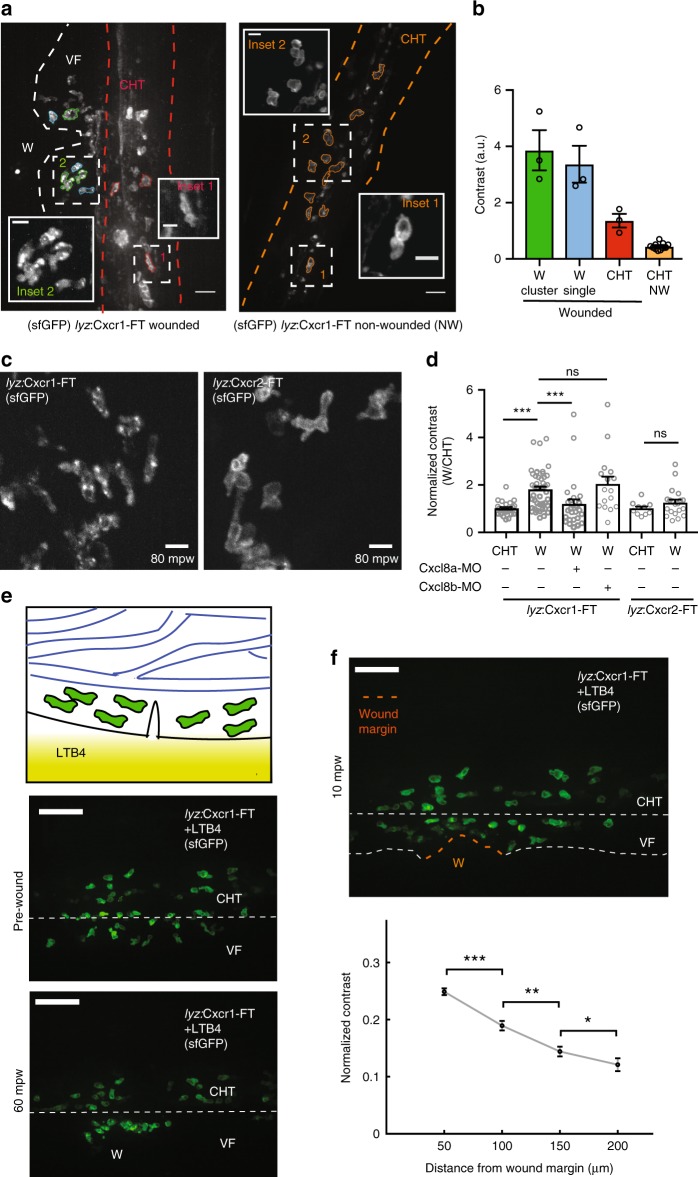
Distinct trafficking of Cxcr1 and Cxcr2 during neutrophil migration to wounds. **a** Overview of receptor distribution patterns and corresponding quantitative approach. Confocal projection of neutrophils in a representative wounded or unwounded Tg(*lyz*:Cxcr1-FT) larva. Examples of cells are shown in different colors: single (blue) or clustered (green) cells at the wound, cells in the CHT of the same wounded larva (red) or cells in the CHT of an unwounded larva (orange). CHT: caudal hematopoietic tissue, VF: ventral fin, W: wound. **b** Calculation of contrast from the cells segmented in **a**. *n* = 3 (green, blue, red), *n* = 11 (orange) cells. Scale bar = 25 µm, scale bar (insets) = 10 µm. **c** Confocal projection of neutrophils in Tg(*lyz*:Cxcr1-FT) or Tg(*lyz*:Cxcr2-FT) larvae at the wound focus. Scale bar = 10 µm. mpw = minutes post wound. **d** Normalized contrast (contrast per individual neutrophil normalized to the mean contrast of non-mobilized cells in the CHT). Cxcl8a refers to injection of a splice-blocking together with a translation-blocking morpholino for *cxcl8a*. *cxcl8b* refers to injection with a splice-blocking morpholino for Cxcl8b. For Tg(*lyz*:Cxcr1-FT): *n* = 24 cells (CHT), *n* = 47 cells (wound) from 8 larvae. For Tg(*lyz*:Cxcr1-FT) with morpholinos: *n* = 28 cells (Cxcl8a-MO) from 5 larvae, *n* = 16 cells (Cxcl8b-MO) from 5 larvae. For Tg(*lyz*:Cxcr2-FT): *n* = 10 cells (CHT) and *n* = 20 cells (wound) from 3 larvae. Data were pooled from independent larvae acquired in 1 to 5 imaging sessions. Kruskal–Wallis test with Dunn’s multiple comparisons test for Tg(*lyz*:Cxcr1-FT), two-tailed unpaired Mann–Whitney test for Tg(*lyz*:Cxcr2-FT). **e** Top: cartoon depicting neutrophils in the CHT after mobilization to the ventral fin in response to an exogenous LTB4 gradient. Bottom: confocal projections of Cxcr1-FT neutrophils 30 min after LTB4 addition, right before (middle) and 1 h after wound (bottom). Scale bar = 50 µm. **f** Same larva as in **e**, at 10 mpw. Orange dashed line shows the wound margin. Graph below shows normalized contrast of individual neutrophils as a function of distance from the nearest point in the wound margin. For normalization, values were divided by the maximum contrast of each movie. *n* > 92 cells or clustered cells per bin, from 3 larvae in 3 imaging sessions. Two-tailed unpaired Mann–Whitney test. Error bars represent S.E.M. across cells. Source data are provided as a Source Data file

To quantify these dynamics, we devised an approach that relied solely on the distribution pattern of sfGFP (Fig. 2a, b). We computed the contrast of the sfGFP signal in surface-segmented neutrophils. This metric reflects intensity differences between neighboring pixels^32^ and is higher in cells with punctate/vesicular distribution of the receptor, as opposed to cells with smooth intensity fluctuations at the plasma membrane (Fig. 2a, b). We found this method more suitable for neutrophils than a ratiometric approach that scores membrane receptor levels over a control membrane marker, as it bypasses the need for accurate membrane segmentation. The latter was difficult to achieve due to the irregular shape and membrane configuration of neutrophils, as well as their dense clustering (Supplementary Fig. 1). Quantification confirmed that Cxcr1-FT, but not Cxcr2-FT, markedly internalized at wounds (Fig. 2c, d).

To establish whether Cxcr1-FT internalization was in response to the putative Cxcr1/2 ligands Cxcl8a or Cxcl8b, we used knockdown of the two chemokines with previously validated morpholinos^33–35^. We found that *cxcl8a* knockdown markedly suppressed the internalization of Cxcr1-FT, whereas *cxcl8b* knockdown was ineffective (Fig. 2d, Supplementary Fig. 1a, b, and Supplementary Movie 5). As further evidence for Cxcl8a-induced internalization, we utilized an ectopic expression system. We injected Cxcr1-FT or Cxcr2-FT mRNA in one-cell-stage embryos to obtain a uniform constitutive expression of the receptors in gastrulating embryos, in which cell membranes are easily visualized and segmented (Supplementary Fig. 3). Both receptors showed membrane distribution comparable to a control membrane enhanced cyan fluorescent protein (membrane ECFP, hereafter referred to as mCFP) (Supplementary Fig. 3a). However, when Cxcl8a was co-expressed, Cxcr1 showed a marked internalization (Supplementary Fig. 3a). Quantification of the ratio of sfGFP (Cxcr1) levels at the plasma membrane over a control mCFP indicated receptor downregulation from the cell surface in the presence of Cxcl8a, which was not observed in the case of Cxcr2 (Supplementary Fig. 3b). These patterns did not arise from a crosstalk between ECFP and sfGFP detection, and were also observed without mCFP co-expression (Supplementary Fig. 4).

To probe for endogenous Cxcr1 internalization in response to Cxcl8a, we visualized neutrophil uptake of a fluorescent fusion of Cxcl8a. We transplanted human embryonic kidney (HEK293T) cells expressing Cxcl8a-mCherry in zebrafish larvae, as previously described and validated^35^. In wild-type Tg(*mpx*:GFP)^i114^ transgenic larvae, we observed a considerable accumulation of Cxcl8a-mCherry inside the neutrophils contacting the transplant (Supplementary Fig. 5 and Supplementary Movie 6). By contrast, in Tg(*mpx*:GFP)^i114^ (*sa14414* is a null mutant allele of *cxcr1*) we observed a significantly reduced Cxcl8a-mCherry uptake. This suggested that endogenous Cxcr1 internalizes in response to Cxcl8a, much like the Cxcr1-FT construct.

Altogether, this evidence showed that Cxcr1, but not Cxcr2, internalizes and is downregulated in response to endogenous Cxcl8a at sites of tissue damage.

### Cxcr1 downregulation reports endogenous Cxcl8a gradients

To characterize the pattern of Cxcl8a distribution, we performed further quantitative analyses. To facilitate quantification of Cxcr1 internalization, we forced neutrophils to enter the ventral fin by applying an alternative chemoattractant, LTB4, in the bath of the larva (Fig. 2e)^36^. This led to a quick entry of neutrophils into the ventral fin providing more cell numbers for quantification and eliminating the contribution of Cxcr1 internalization of cells in the CHT (Fig. 2e and Supplementary Movie 7). Neutrophils did not show chemokine internalization after LTB4 treatment, further confirming specificity of Cxcr1 internalization in response to Cxcl8a (Supplementary Movie 7). After performing a wound, we quantified internalization of Cxcr1 as a function of the distance from the closest point of the wound margin (Fig. 2f). Quantification reported a gradient of a range of 200 µm (Fig. 2f).

### Cxcr1 and Cxcr2 recognize Cxcl8a and Cxcl8b, respectively

The differential trafficking behavior of Cxcr1 and Cxcr2 could have been due to differential ligand preferences of the receptors and/or differential trafficking fates in response to the corresponding ligands. A biochemical characterization of the ligand preferences of Cxcr1 and Cxcr2 was lacking. To elucidate the ligand preferences of the receptors, we used a reductionist approach. We transfected mammalian HEK293T cells with plasmids coding for Cxcr1 or Cxcr2. We then employed label-free live-cell biosensing based on a dynamic mass redistribution (DMR) to characterize receptor function in response to individually applied ligands (Cxcl8a and Cxcl8b) (Supplementary Fig. 6a). This technique measures integrated whole-cell responses of living cells to pharmacological stimuli.^37,38^. Cxcr1, but not Cxcr2-expressing HEK293T cells, showed specific DMR responses when exposed to recombinant Cxcl8a or supernatant prepared from Cxcl8a-transfected HEK293T cells (Supplementary Fig. 6a). By contrast, activation of Cxcr2 but not of Cxcr1 occurred only when transfectants were exposed to Cxcl8b-containing supernatants (Supplementary Fig. 6a). This indicated that Cxcr1 and Cxcr2 recognize Cxcl8a and Cxcl8b, respectively, at least in isolation from each other.

This finding contrasted previous genetic experiments showing that Cxcr2 knockdown compromises responses to Cxcl8a injection in vivo^36^, which had suggested a possible ligand–receptor interaction. To reconcile the two findings, we interrogated the dependence of neutrophil Cxcl8a-driven chemotaxis on Cxcr1 and Cxcr2 in a reductionist in vitro setting. We used an in vitro transwell chemotaxis assay using neutrophils from wild-type Tg(*mpx*:GFP)^i114^, Tg(*mpx*:GFP)^i114^/*cxcr1*^*sa14414/sa14414*^ (*cxcr1*^*−/−*^ hereafter *sa6118* is a null mutant allele of *cxcr2*), and Tg(*mpx*:GFP)^i114^/*cxcr2*^*sa6118/sa6118*^ (*cxcr2*^*−/−*^ hereafter; *sa6118* is a null mutant allele of *cxcr2*) zebrafish (Supplementary Fig. 6b, c). Wild-type neutrophils transmigrated in response to Cxcl8a but both *cxcr1*^*−/−*^ and *cxcr2*^*−/−*^ neutrophils showed a markedly reduced response (Supplementary Fig. 6d). Notably, *cxcr1*^*−/−*^ neutrophils showed no residual response, whereas *cxcr2*^*−/−*^ neutrophils showed a modest residual response. Together, this evidence confirmed the genetic dependency of Cxcl8a responses on Cxcr2 but indicated that this does not arise from a direct ligand–receptor interaction.

The revised ligand preferences raised the question whether lack of Cxcr2 internalization in neutrophils at wounds was due to the absence of Cxcl8b in ventral fin wounds or inability to internalize in response to this ligand. Reverse transcription polymerase chain reaction (RT-PCR) analysis from ventral fin-wounded larvae revealed an upregulation of both Cxcl8a and Cxcl8b by 30 min after wounding, suggesting both ligands are likely present (Supplementary Fig. 6e). To assess the internalization of Cxcr2 in response to Cxcl8b, we used ectopic expression in gastrulating embryos (Supplementary Fig. 7). Cxcr2 was internalized in the presence of Cxcl8b (Supplementary Fig. 7a). Quantification did not capture a significant downregulation of the receptor from the plasma membrane (Supplementary Fig. 7b), suggesting either a limitation in the sensitivity of this metric or possible differences in the internalization pattern of Cxcr1 and Cxcr2.

To therefore clarify possible differences in the internalization pattern of Cxcr1 and Cxcr2 in response to their corresponding ligands, we switched to a higher-resolution in vitro system that allowed acute, short-term exposure to cognate ligands, which better reflected the timescale of ligand exposure in neutrophils in vivo. To this end, we expressed Cxcr1-FT and Cxcr2-FT in HEK293T cells in vitro. Visualization of Cxcr1-FT and Cxcr2-FT 15 or 30 min after exposure to Cxcl8a and Cxcl8b, respectively, revealed distinct intracellular localizations. sfGFP-positive vesicles in Cxcr2-FT-expressing HEK293T cells were localized along the plasma membrane, whereas the equivalent vesicles were localized deeper in the cytosol in Cxcr1-FT-expressing cells (Supplementary Fig. 7c). Altogether, these data suggested that Cxcr1 and Cxcr2 both internalize in response to their corresponding ligands but the former does so in a manner consistent with downregulation, whereas the latter does so in a manner consistent with recycling.

### Distinct roles of Cxcr1 and Cxcr2 in neutrophil dispersal

We then investigated the functional contributions of Cxcr1 and Cxcr2 in the migratory response on neutrophils to ventral fin wounds, with a view to linking receptor trafficking behavior with receptor function. A previous study using null mutants of Cxcr1 and Cxcr2, photoconvertible reporters, and a tail fin wound model showed different roles for Cxcr1 and Cxcr2 in forward and reverse traffic^11^. Here we sought to characterize two independent knockout lines for these receptors and to develop analysis methods to distinguish forward and reverse traffic defects in real-time trajectory data. To this end, we took advantage of the ventral fin wound model, which has accelerated kinetics, facilitating the tracking of reverse migration in real time. Here, maximal neutrophil accumulation occurs as early as 1–2 h post wound (hpw) (as opposed to 6 h in a tail fin wound), due to the proximity of the target wound site to the starting location of the cells (CHT).

**Fig. 3:**
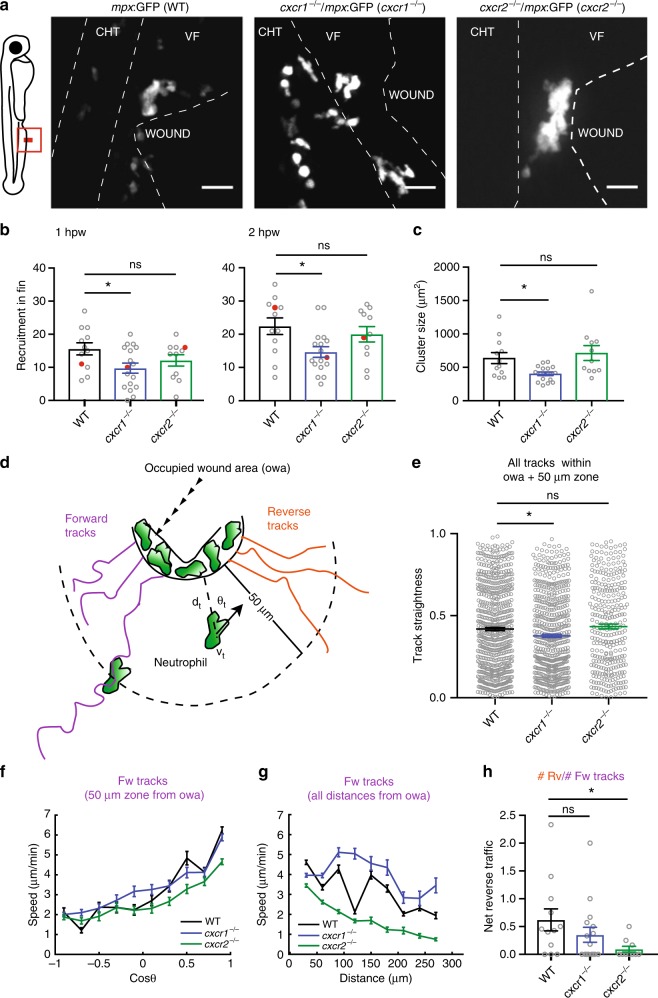
Differential contributions of Cxcr1 and Cxcr2 in neutrophil clustering and dispersal. **a** Confocal projections showing distribution of neutrophils at wounds of wild-type (WT) Tg(*mpx*:GFP)^i114^, *cxcr1*^*−/−*^ (*cxcr1*^*−/−*^/Tg(*mpx*:GFP)^i114^), or *cxcr2*^*−/−*^ (*cxcr2*^*−/−*^/Tg(*mpx*:GFP)^i114^) larvae at 2 hpw. CHT: caudal hematopoietic tissue, VF: ventral fin. Cartoon on the left indicates area imaged. Dashed lines show VF and CHT outlines. Scale bar = 25 µm. **b** Number of recruited neutrophils at 1 and 2 hpw, within a square area of 200 × 200 µm around the wound. One-way ANOVA with Tukeyʼs multiple comparisons test. *n* = 12 (WT), *n* = 17 (*cxcr1*^*−/−*^), and *n* = 11 (*cxcr2*^*−/−*^) larvae. Larvae shown in **a** are represented with a red dot. **c** Average neutrophil cluster size per larva. *n* = 12 (WT), *n* = 17 (*cxcr1*^*−/−*^), and *n* = 11 (*cxcr2*^*−/−*^) larvae. Kruskal–Wallis test with Dunn’s multiple comparisons test. **d** Cartoon depicting trajectory parameters measured. The occupied wound area (owa) is the area occupied by the neutrophil cluster. Forward (magenta) and reverse (orange) segments of cell trajectories are defined as the path of neutrophils prior to entering and after leaving the owa, respectively. *d*_t_, shortest distance from owa at time point *t*. *v*_t_, speed at time point *t*. *θ*_t_ = approach angle to owa at time point *t*. **e** Neutrophil track straightness within the owa and an area extending 50 µm beyond. *n* = 680 tracks (WT), *n* = 603 tracks (*cxcr1*^*−/−*^), and *n* = 319 tracks (*cxcr2*^*−/−*^). Kruskal–Wallis test with Dunn’s multiple comparisons test. **f** Neutrophil speed in relation to the cosine of the angle *θ*, within a zone of 0–50 µm from the owa are shown. *n* = 131–2423 steps per bin (WT), *n* = 11–3008 steps per bin (*cxcr1*^*−/−*^), *n* = 88–2823 steps per bin (*cxcr2*^*−/−*^). **g** Neutrophil speed in relation to distance from the owa. *n* = 133–1227 cell steps per bin (WT), *n* = 231–1436 steps per bin (*cxcr1*^*−/−*^), *n* = 202–1382 steps per bin (*cxcr2*^*−/−*^). **h** Net reverse traffic. *n* = 12 (WT), *n* = 17 (*cxcr1*^*−/−*^), and *n* = 11 (*cxcr2*^*−/−*^) larvae. In all panels, data are from the same 12 WT, 17 *cxcr1*^*−/−*^, and 11 *cxcr2*^*−/−*^ larvae from 6, 10, and 8 imaging sessions, respectively. Cells were analyzed from the start of the movie (~15 mpw) up to 2 hpw. Error bars represent S.E.M. across cell steps (f,g) or cell tracks (e) or larvae (b,c,h). Source data are provided as a Source Data file

We imaged neutrophil behavior in ventral fin wounds of wild-type, *cxcr1*^*−/−*^ and *cxcr2*^*−/−*^ zebrafish in a Tg(*mpx*:GFP)^i114^ background. As part of a general validation of our knockout lines, we measured neutrophil distribution and numbers in the absence of wounding and found these to be comparable in the knockouts compared with wild-type zebrafish (Supplementary Fig. 8). Consistent with previous reports, we found that Cxcr1 and Cxcr2 have different roles in neutrophil migration behavior (Supplementary Movie 8). Neutrophils in wild-type Tg(*mpx*:GFP)^i114^ embryos showed clustering at the wound but also some degree of exploration and dispersal (Fig. 3a and Supplementary Movie 8). Neutrophils in Tg(*mpx*:GFP)^i114^/*cxcr1*^*−/−*^ larvae showed reduced recruitment and cluster size (Supplementary Movie 8 and Fig. 3a, b, c). On the other hand, neutrophils in Tg(*mpx*:GFP)^i114^/*cxcr2*^*−/−*^ larvae showed reduced dispersal (Supplementary Movie 8).

To quantify forward and reverse migration defects, we developed a protocol that bypasses the need for intra-cluster tracking accuracy. We generated a code that classifies tracks as forward and reverse tracks. Tracks were classified as “forward” if the start position was outside the occupied wound area (owa; i.e., the area occupied by clustering neutrophils, ranging 50–70 µm from the wound margin) and the cells intersected with the owa thereafter (datapoints after intersection with the owa were excluded) (Fig. 3d). In reverse tracks, data within the owa and before entering the owa were excluded (Fig. 3d). Statistics could then be performed on forward and reverse motion separately or on pooled tracks. To probe chemotaxis differences, we measured track straightness of pooled tracks within the area covering the owa and a zone extending of 50 µm from the owa, which corresponded to the most significant part of the chemokine gradient (Fig. 2f, 0–150 µm from wound margin). Neutrophils in Tg(*mpx*:GFP)^i114^/*cxcr1*^*−/−*^ larvae showed reduced track straightness in this area (Fig. 3e). When assessing the forward movement, we found that forward tracks showed a comparable bias on directional speed between the three conditions (Fig. 3f), as measured by a trend for higher speeds when neutrophils were oriented towards the target. Conversely, neutrophils in Tg(*mpx*:GFP)^i114^/*cxcr2*^*−/−*^ larvae showed a reduced speed independently of orientation and across a range of distances (Fig. 3f, g). This indicated that neutrophils in Tg(*mpx*:GFP)^i114^/*cxcr2*^*−/−*^ are slower to reach the wound, and that Cxcr2 signaling contributes to the forward traffic. However, consistent with observations using photoconvertible reporters^11^, the most prominent phenotype of Tg(*mpx*:GFP)^i114^/*cxcr2*^*−/−*^ neutrophils was the reduced dispersal from the wound, as indicated here by a lower ratio of reverse over forward tracks (net reverse traffic; Supplementary Movie 8 and Fig. 3h). Follow-up staining of neutrophils at a late stage of 24 hpw confirmed a defect in resolution in these mutant larvae (Supplementary Fig. 9).

Altogether, these results revealed the specific functions of the two receptors and validated our quantitative analysis approach. These results also functionally showed that the Cxcr2 ligand(s) is (are) likely present in ventral fin wounds.

### Cxcr1 and Cxcr2 have redundant roles in wound recruitment

Previous studies, using a chemical inhibitor of human CXCR2, did not reveal a role in neutrophil dispersal but rather in recruitment to wounds^33,34^. We hypothesized that the Cxcr1/Cxcl8a and the Cxcr2/Cxcl8b signaling pathways might be partly redundant in recruiting neutrophils to the wound and compensating for each other in *cxcr2*^*−/−*^ zebrafish. To explore this, we assessed the behavior of neutrophils in wounds under combinatorial Cxcr1 and Cxcr2 inhibition. Cxcr1 and Cxcr2 are adjacently located on the same chromosome precluding the generation of double knockout fish by crossing. We thus injected a previously validated morpholino against Cxcr2^36^ into *cxcr1*^*−/−*^ larvae. In these fish, we detected a marked defect in recruitment compared with *cxcr1*^*−/−*^ and wild-type larvae (Supplementary Movie 8 and Supplementary Fig. 10a, b). The relatively few neutrophils that were recruited showed no directional bias on speed and markedly lower speed levels during forward traffic (Supplementary Fig. 10c, d). To ascertain that the Cxcr2 morpholino did not cause off-target effects, we compared its effect in *cxcr1*^*−/−*^ and *cxcr2*^*−/−*^ larvae. We found that the morpholino reduced the overall accumulation of neutrophils in tail fin wounds in *cxcr1*^*−/−*^ but not in *cxcr2*^*−/−*^ larvae, confirming the specificity of targeting (Supplementary Fig. 10e, f). These results clarified that both Cxcr1 and Cxcr2 contribute to the forward traffic but these functions are largely redundant, whereas their respective roles in clustering and in dispersal are non-redundant.

### Receptor mutations alter Cxcr1 and Cxcr2 trafficking

**Fig. 4:**
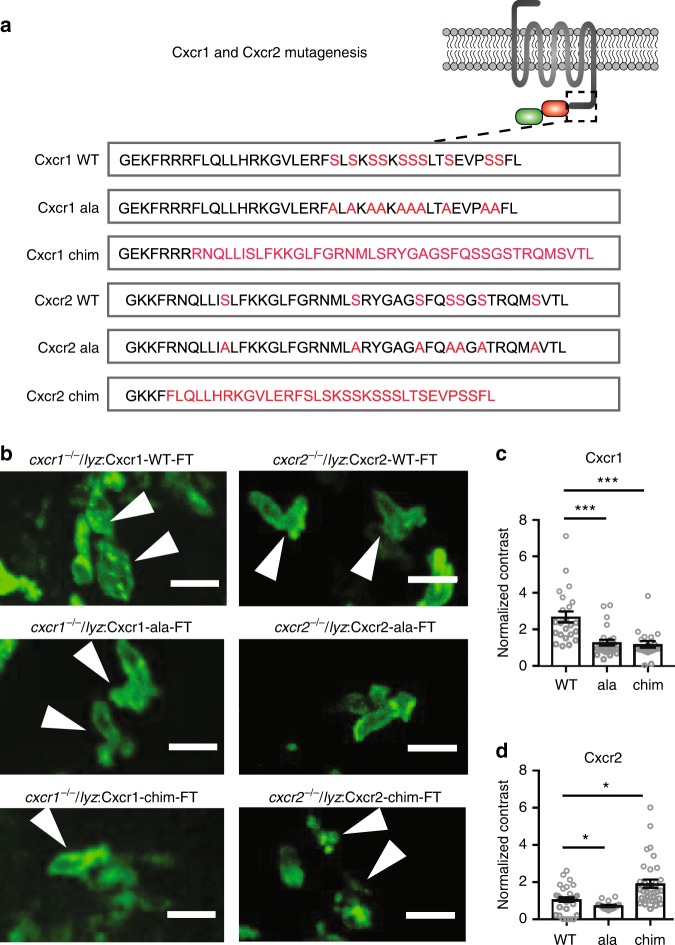
Receptor mutagenesis alters Cxcr1 and Cxcr2 trafficking. **a** Mutagenesis of Cxcr1. Amino acid sequence of the C-terminus is shown with candidate phosphorylation targets (serines) or transplanted sequences highlighted in red. **b** Neutrophils in *cxcr1*^*−/−*^/*cxcr2*^*−/−*^ larvae rescued by transgenic neutrophil-specific expression of Cxcr1-FT/Cxcr2-FT, Cxcr1-ala-FT/Cxcr2-ala-FT, or Cxcr1-chim-FT/Cxcr2-chim-FT receptors. Arrows point to neutrophils at the center of the wound, with representative distribution of the receptor. Scale bar = 15 µm. **c** Quantification of contrast in *cxcr1*^*−/−*^ or *cxcr1*^*−/+*^ neutrophils rescued by the different Cxcr1-FT receptor variants. *n* = 23 cells (WT) from 7 larvae, *n* = 26 cells (ala) from 6 larvae, *n* = 19 cells (chim) from 5 larvae. Data were acquired in 2 to 4 imaging sessions. Kruskal–Wallis test with Dunn’s multiple comparisons test. **d** Quantification of contrast in *cxcr2*^*−/−*^ neutrophils rescued by the different Cxcr1-FT receptor variants. *n* = 33 cells (WT) from 5 larvae, *n* = 18 cells (ala) from 5 larvae, *n* = 33 cells (chim) from 8 larvae. Data are from independent larvae in 3 to 5 imaging sessions. Kruskal–Wallis test with Dunn’s multiple comparisons test. Error bars represent S.E.M. across cells. Source data are provided as a Source Data file

We found that Cxcr1 and Cxcr2 have distinct trafficking in neutrophils at wound sites despite the likely presence of the corresponding endogenous ligands in this context. This raised the question whether the receptors have intrinsic differences in ligand-induced trafficking. To test this, we used mutagenesis of Cxcr1 and Cxcr2. GPCR downregulation is determined by a cluster of serine residues at the C-terminus of the receptor that are substrates for GRK phosphorylation and recruit β-arrestins with high affinity^14–16^. We noted that the C-terminus of Cxcr1 carries a higher incidence of adjacently located serine residues in comparison with Cxcr2 (Fig. 4a). We generated a mutant of Cxcr1 where all the C-terminal serines are replaced with alanines (Cxcr1-ala; Fig. 4a) and a mutant whereby the Cxcr1 C-terminus is replaced with that of Cxcr2 (Cxcr1-chim) (Fig. 4a). We generated equivalent constructs for Cxcr2 (Fig. 4a).

We then produced rescue transgenic lines where either a wild-type Cxcr1-FT (Cxcr1-WT), a Cxcr1-FT-ala (Cxcr1-ala), or a Cxcr1-FT-chimera (Cxcr1-chim) was expressed in neutrophils of *cxcr1*^*−/−*^ fish or a wild-type Cxcr2-FT (Cxcr2-WT), a Cxcr2-FT-ala (Cxcr2-ala) or a Cxcr2-FT-chimera (Cxcr2-chim) was expressed in neutrophils of *cxcr2*^*−/−*^ fish. We retained usage of the FT cassette, instead of switching to sfGFP single fusion, despite the limited usage of tagRFP in our analyses. This was to keep a consistency with the analyses from the Tg(*lyz:*Cxcr1-FT) and Tg(*lyz:*Cxcr2-FT) zebrafish, and to facilitate screening of transgenic embryos and cell tracking based on the brighter signal of tagRFP.

As anticipated, the Cxcr1-ala mutant receptor showed membranous distribution in neutrophils at wounds, consistent with an inability to desensitize (Fig. 4b, c and Supplementary Movie 9). The chimeric mutant receptor (Cxcr1-chim) was also sustained at the neutrophil cell membrane, indicating that plasma membrane sustenance of Cxcr2 was transferable to Cxcr1 through its C-terminus (Fig. 4b, c and Supplementary Movie 9). The Cxcr2-ala mutant receptor showed membranous distribution, whereas the Cxcr2-chim was markedly internalized in neutrophils at wound sites (Fig. 4b, c and Supplementary Movie 10). This indicated that the relatively low extent of internalization of Cxcr2 at wounds compared with Cxcr1 was due to the lack of appropriate motifs in the cytoplasmic tail of the receptor. To confirm that Cxcr2-chim internalization is Cxcl8b-dependent at wounds, we used Cxcl8b morpholino treatment. Cxcl8b morphants showed reduced internalization in comparison with non-treated Cxcr2-chim neutrophils, consistent with the ligand preference of this receptor (Supplementary Fig. 11 and Supplementary Movie 11). To further confirm that Cxcr2-chim recognizes Cxcl8b, we expressed the receptor in early gastrulating embryos with or without Cxcl8b (Supplementary Fig. 11). Cxcr2-chim showed significant internalization in response to Cxcl8b but not in response to Cxcl8a (Supplementary Fig. 12). These results confirmed that Cxcr2-chim recognizes the same ligand as Cxcr2. We were not able to reconstitute expression of the chimeric receptors in HEK293T cells to compare the trafficking pattern of Cxcr2-chim with Cxcr2-WT. However, altogether, the evidence from neutrophils suggested that Cxcr2-chim has enhanced downregulation in response to Cxcl8b in wounds in comparison with Cxcr2-WT.

### Dispersal requires Cxcr2 sustenance at the plasma membrane

**Fig. 5:**
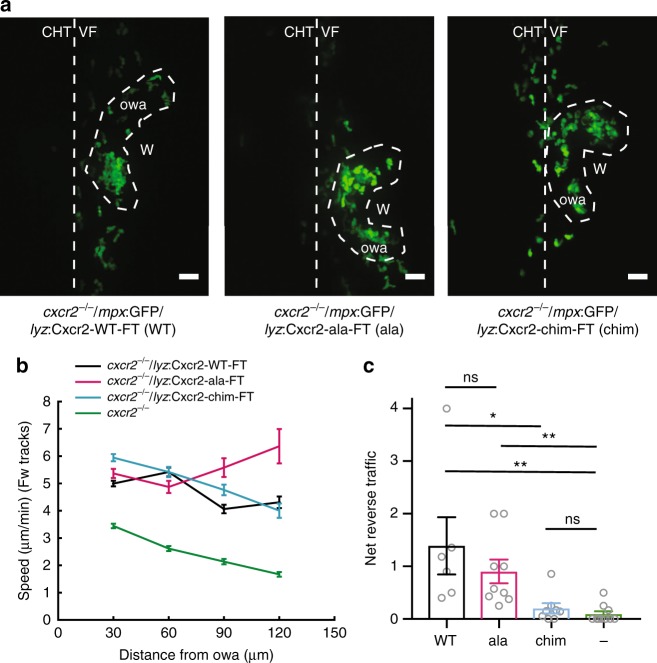
Plasma membrane sustenance of Cxcr2 is required for neutrophil dispersal. **a** Confocal projections of neutrophil distribution in Tg(*lyz:*Cxcr2-WT-FT)*/cxcr2*^*−/−*^ larvae, Tg(*lyz:*Cxcr2-ala-FT)*/cxcr2*^*−/−*^, and Tg(*lyz:*Cxcr2-chim-FT)*/cxcr2*^*−/−*^ at ~2 hpw. Dashed line indicates occupied wound area (owa). CHT: caudal hematopoietic tissue, VF: ventral fin, W: wound. Scale bar = 32 µm. **b** Neutrophil speed in relation to distance from the owa. Average speeds per cell per distance bin are shown. *n* = 558–2651 steps per bin (WT), *n* = 95–1266 steps per bin (ala), *n* = 494–2000 steps per bin (chim), and *n* = 1168–2823 steps per bin for (*cxcr2*^*−/−*^). **c** Net reverse traffic. *n* = 6 (WT), *n* = 9 (ala), *n* = 8 (chim), *n* = 11 (−; *cxcr2*^*−/−*^) larvae. Kruskal–Wallis test with Dunn’s multiple comparisons test. In **b** and **c**, data are from 6 (WT), 9 (ala), 8 (chim), and 11 (−; *cxcr2*^*−/−*^) larvae from 3, 4, 3, and 8 imaging sessions, respectively. Cells were analyzed from the start of the movie (~15 mpw) up to 2 hpw. Error bars represent S.E.M. across cell steps (b) or larvae (c). Source data are provided as a Source Data file

The above findings indicated that Cxcr1 and Cxcr2 have distinct trafficking responses to their corresponding ligands at wounds. To causally link receptor trafficking with the distinct functions of the receptors in neutrophil migration behavior, we tracked neutrophil behavior in the receptor rescue lines. We first analyzed neutrophil behavior in ventral fin wounds in the Cxcr2 rescue lines (Fig. 5a). Interestingly, all Cxcr2 receptor constructs (WT, Cxcr2-ala and Cxcr2-chim) rescued the slow forward motility defect of *cxcr2*^*−/−*^ neutrophils (Fig. 5b). Both Cxcr2-WT and Cxcr2-ala also rescued the dispersal defect of the *cxcr2*^*−/−*^ neutrophils (Fig. 5c and Supplementary Movie 12). However, the Cxcr2-chim receptor was unable to rescue the dispersal defect (Fig. 5c and Supplementary Movie 12). This demonstrated that sustained residence at the plasma membrane is specifically required for reverse migration but not for forward migration.

### Receptor internalization limits neutrophil motion at wounds

The analysis of Cxcr2 receptor mutants raised the question how receptor plasma membrane sustenance supports dispersal from the target site. To this end, we assessed the functional impact of Cxcr1 mutations that render the receptor resistant to internalization (Cxcr1-ala and Cxcr1-chim). Both these receptor mutants showed a gain of Cxcr1 plasma membrane sustenance. Thus, any shared gain of functions on cell migration behavior would point to the functional importance of plasma membrane sustenance.

**Fig. 6:**
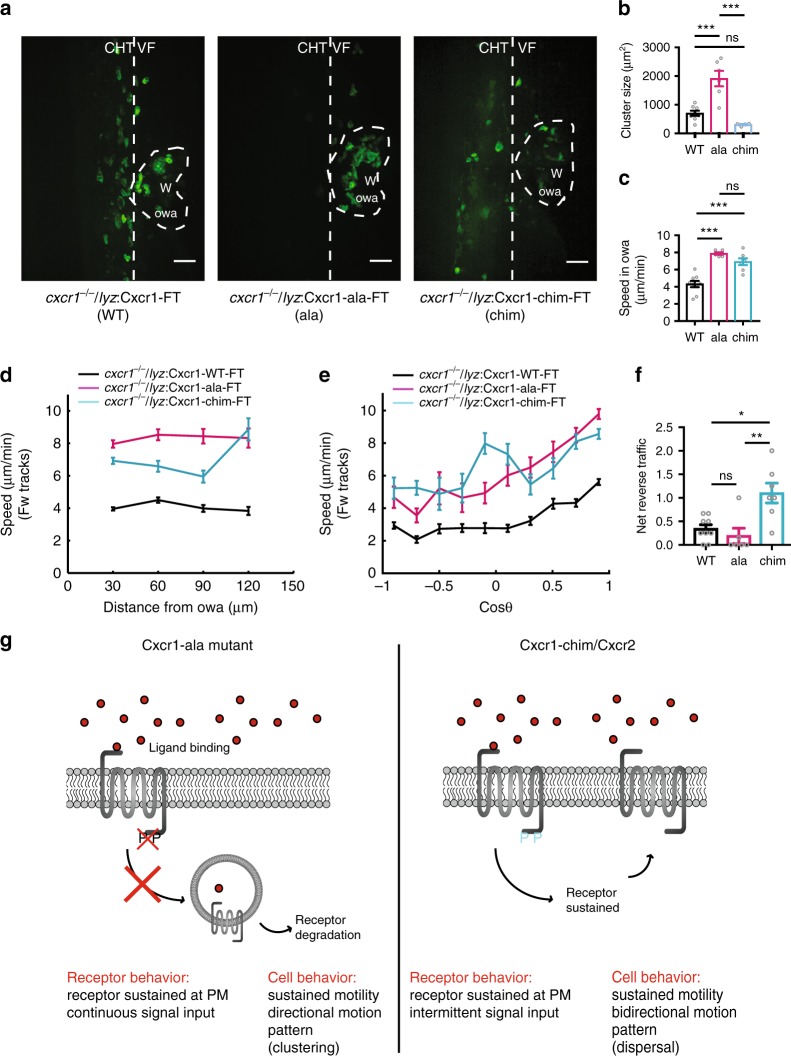
Receptor internalization limits neutrophil motion at wounds. **a** Confocal projections of neutrophil distribution in Tg(*lyz:*Cxcr1-WT-FT)*/cxcr1*^*−/−*^ larvae (WT), Tg(*lyz:*Cxcr1-ala-FT)*/cxcr1*^*−/−*^ (ala), Tg(*lyz:*Cxcr1-chim-FT)*/cxcr1*^*−/−*^(chim) at ~2 hpw. Dashed line indicates occupied wound area (owa). CHT: caudal hematopoietic tissue, VF: ventral fin, W: wound. Scale bar = 32 µm. **b** Quantification of neutrophil cluster size, *n* = 8 (WT), *n* = 6 (ala), and *n* = 4 (chim) larvae from 3 imaging sessions per condition. One-way ANOVA test with Tukeyʼs multiple comparisons test. **c** Quantification of speed within the owa. *n* = 9 (WT), *n* = 6 (ala), and *n* = 7 (chim) larvae. One-way ANOVA test with Tukeyʼs multiple comparisons test. **d** Neutrophil speed in relation to distance from the owa. Average speeds per cell step per distance bin are shown. *n* = 316–1942 steps per bin (WT), *n* = 105–706 steps per bin (ala), *n* = 83–896 steps per bin (chim). **e** Neutrophil speed in relation to cosine of angle *θ*. Average speeds per cell per cos*θ* bin are shown. *n* = 128–849 steps per bin (WT), *n* = 22–445 steps per bin (ala), and *n* = 44–417 steps per bin (chim). **f** Net reverse traffic. *n* = 9 (WT), *n* = 6 (ala), and *N* = 7 (chim) larvae. Kruskal–Wallis test with Dunn’s multiple comparisons test. **g** Summary of phenotypes observed in Cxcr1 rescue experiments and their interpretation. PM, plasma membrane. For **c**–**f**, data are from 9 (WT), 6 (ala), and 7 (chim) larvae from 3, 6, and 3 imaging sessions, respectively. For all panels, analysis was focused on the post initial arrival window of 1–2 hpw. Error bars represent S.E.M. across cell steps (d,e) or larvae (b,c,f). Source data are provided as a Source Data file

We visualized neutrophil migration behavior at wound sites in rescue lines of Cxcr1-WT, Cxcr1-ala, and Cxcr1-chim expressed in a *cxcr1*^*−/−*^ background. The Cxcr1-WT receptor rescued the clustering of *cxcr1*^*−/−*^ neutrophils and the modest differences in speed observed in this mutant with respect to wild-type neutrophils (Supplementary Movie 13 and Supplementary Fig. 13). Cxcr1-ala-expressing neutrophils showed markedly enhanced clustering compared with Cxcr1-WT neutrophils, whereas Cxcr1-chim-expressing neutrophils formed clusters of comparable size to Cxcr1-WT-expressing neutrophils. Despite these differences, the Cxcr1-ala and Cxcr1-chim shared a gain of function in speed of motion, both inside the owa (Fig. 6c) as well as throughout their forward migration to the owa (Fig. 6d, e). This indicated that plasma membrane sustenance sustains motility input in vivo. In addition, these results indicated that the enhanced clustering of Cxcr1-ala-expressing mutants was not due to enhanced stopping at the owa but to a gain of unidirectional, focalized motion pattern that favored congregation (see also Supplementary Movie 13). By contrast, the Cxcr1-chim-expressing mutant neutrophils showed a gain of exploratory motion (Supplementary Movie 13), which was less directionally biased (Fig. 6e) and which resulted in enhanced dispersal (Fig. 6f) with respect to Cxcr1-WT-expressing neutrophils.

Altogether, this evidence revealed the role of receptor sustenance in sustaining neutrophil motility in vivo.

### Distinct temporal signaling profiles of Cxcr1 and Cxcr2

The differences in motion pattern observed between Cxcr1-ala and Cxcr1-chim suggested that plasma membrane sustenance is not the only determinant in Cxcr2-mediated dispersal and other signaling factors, linked to the C-terminus of Cxcr2, must be involved in interpreting the external gradient into bidirectional motion. To obtain insight into signal processing differences, we switched to reductionist DMR studies in HEK293T cells. We explored possible differences in temporal signal processing and how these may depend on the phosphorylation status of the receptor. Cxcr1 and Cxcr2 showed temporal signaling profiles consistent with their differential trafficking. Cxcr1 showed a prominent signaling response upon first exposure to the ligand (Cxcl8a), followed by slow decay and an attenuated response to secondary exposure (Supplementary Fig. 14a). By contrast, Cxcr2 showed a rapid signal decay after stimulation (by Cxcl8b) and an ability to be re-stimulated to maximal levels upon secondary exposure (Supplementary Fig. 14a). Both Cxcr1-ala and the Cxcr2-ala mutants showed more sustained responses after the first stimulation, consistent with the plasma membrane sustenance of both these receptors (Supplementary Fig. 14a, b). However, Cxcr2-ala retained a trend for a more discrete and potent response upon second stimulation, which was not seen in the Cxcr1-ala receptor. This suggested additional phosphorylation-independent differences in temporal signal processing. We explored whether such differences might stem from differential Gα protein coupling preferences for Cxcr1 and Cxcr2. Mammalian neutrophil chemoattractant receptors can couple to Gi or Gq subunits^39^. We therefore tested receptor coupling to these subunits using well-defined pharmacological treatments. Cxcr1 and Cxcr2 showed comparable coupling to Gi and Gq, as their DMR ligand-induced responses could be suppressed by the corresponding inhibitors (pertussis toxin (PTX) and FR900359^40^) (Supplementary Fig. 15a, b). These coupling profiles were not changed by the addition of fluorescent protein fusions or the alanine mutations (Supplementary Fig. 15c, d). Thus, the phosphorylation-independent differences in temporal signal processing are unlikely to arise from differential Gα -coupling profiles.

Altogether, this evidence provided a framework to interpret the different neutrophil motion patterns induced by Cxcr1-chim and Cxcr1-ala in vivo. Cxcr1-chim can be inferred to have the signaling pattern of Cxcr2-WT, with interruptions between signaling rounds, and this intermittent profile correlates with a bidirectional motion that favors dispersal (Fig. 6g). By contrast, the Cxcr1-ala mutations, cause continuous signaling at nearly maximal levels and this profile correlates with unidirectional/focalized motion that supports clustering (Fig. 6g).

## Discussion

**Fig. 7:**
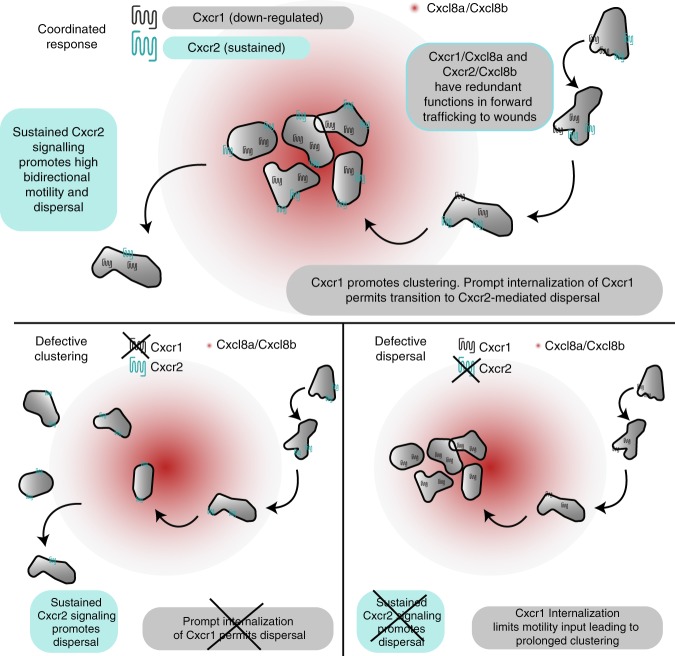
Model for coordination of neutrophil clustering and dispersal through chemokine receptor trafficking. (Top) Cxcr1/Cxcl8a and Cxcr2/Cxcl8b can partly compensate for each other during initial chemotaxis to the wounded tissue. However, Cxcr1 specifically promotes clustering and this contribution is limited by desensitization and downregulation. Conversely, Cxcr2 is recycled after internalization and promotes persistent bidirectional motility in the wounded tissue through sustained, intermittent signaling. This facilitates dispersal from the site. Bottom left: in the absence of Cxcr1, neutrophils are recruited, through Cxcr2/Cxcl8b and other endogenous signals, but show a loss in clustering. Bottom right: in the absence of Cxcr2, neutrophils are recruited, through Cxcr1/Cxcl8a and other endogenous signals. Once at the target, Cxcr1 is maximally downregulated and neutrophils lack signal input for motility, leading to a defect in dispersal

Immune cells utilize multiple chemoattractant receptors to guide themselves to inflammatory sites. How these cells coordinate signal inputs to generate complex migration patterns in vivo has remained a challenge to understand. Here we elucidate how two chemokine receptors fine-tune neutrophil clustering and dispersal at sites of tissue damage (Fig. 7). Cxcr1 promotes clustering of zebrafish neutrophils, whereas Cxcr2 promotes dispersal. These functional contributions arise, in large part, from the distinct sub-cellular trafficking of the two receptors in response to their corresponding ligands in situ. Cxcr1 is downregulated from the plasma membrane in the neutrophils as they cluster at wound sites, leading to a loss of input from this receptor and transition to Cxcr2 signaling. The latter receptor promotes bidirectional motion and is sustained at the plasma membrane. This ensures long-lived chemokinesis, dispersal, and resolution (Fig. 7). Our findings have general implications, as human chemoattractant receptors also exhibit differential ligand-induced trafficking in vitro^24,41^ and the implications of this on immune responses remain elusive.

We used biosensors and quantitative analyses to describe the dynamics of chemokine receptors in live neutrophils in vivo. We found that the constitutive turnover of chemokine receptors is rapid in neutrophils in comparison with other cell types (our paper and ref. ^27^). Thus, fluorescent sensors with a long maturation time may not be suitable to capture receptor dynamics in this cell type. The origin of such cell-type-based differences is unclear, as the constitutive trafficking of chemokine receptors is less characterized than ligand-induced trafficking. In other GPCRs, constitutive internalization depends on receptor motifs and endocytic pathways distinct from those driving agonist-dependent internalization^42^. Developmental differences in these pathways between neutrophils and other cells may exist and this could have implications in signaling. For example, a high constitutive turnover of chemokine receptors might somewhat resist ligand-induced downregulation, increasing the dynamic range of signaling without compromising sensitivity to ligand. This may reflect an evolutionary adaptation to cope with a large range of signal inputs. Internalization of Cxcr1 reported signaling gradients of a range of 200 µm, a somewhat longer range than the reported ectopic Cxcl8a gradients^35^ in zebrafish and endogenous CCL21 chemokine gradients in mice^23,43^. This could reflect differences in ligand distribution in the different settings and/or a higher sensitivity of the readout used here for chemokine levels. The internalization of Cxcr1 upon mobilization of neutrophils from the CHT is consistent with the reported roles of inflammatory chemokines in mobilizing neutrophils from hematopoietic sites^34,36,44^.

We characterized the in vivo trafficking patterns and the ligand preferences of Cxcr1 and Cxcr2. We dissected the implications of receptor trafficking from ligand aspects through our receptor mutagenesis approach. Our functional data conclude on a model whereby Cxcr1 is downregulated in response to Cxcl8a at the wound, whereas Cxcr2 is relatively sustained in response to Cxcl8b likely through a difference in recycling potency. Our findings do not exclude the contribution of other ligands but do clarify that Cxcr1 and Cxcr2 have different agonist preferences. This leads to reinterpretation of previous studies showing that neutrophil responses to Cxcl8a are Cxcr2-dependent in vivo^36^. We confirmed this dependency here, but our evidence suggests that this does not arise from a direct ligand/receptor interaction. Possible mechanisms include formation of a high affinity Cxcr1/2 heterodimer and modulation of Cxcr1 expression and/or function by Cxcr2. On the contrary, the reported role of Cxcr2/Cxcl8b in neutrophil mobilization into the blood stream^33,34^ likely reflects a direct ligand–receptor interaction. Previous studies reported a mild defect for *cxcl8a*^*−/−*^ neutrophils in dispersal^11^, which had also alluded to a direct ligand–receptor interaction for Cxcl8a/Cxcr2. In light of our findings, a possible reinterpretation of this phenotype could be that genetic elimination of Cxcl8a affects Cxcr2/Cxcl8b functionality/expression. It is noteworthy that neither Cxcl8a or Cxcl8b morpholino-mediated knockdown showed a dispersal phenotype in two independent studies^33,34^. This is consistent with the idea that both Cxcl8a/Cxcr1 and Cxcl8b/Cxcr2 axes are involved in forward traffic, and that compensatory effects may be occuring under genetic elimination experiments, but not under knockdown conditions, which may be important for manifestation of dispersal phenotypes.

We elucidated the role of chemokine receptor trafficking on neutrophil migration behavior at inflammatory sites. We show that plasma membrane sustenance of Cxcr2 is important for Cxcr2-mediated dispersal from the wound, as this allows prolonged bidirectional motility on site. This is an important mechanistic insight of broad relevance, as previously it remained unknown why certain receptors can support dispersal/reverse migration and others cannot. Our evidence further suggests that the route via which the receptor is sustained has implications in gradient interpretation. The rapid signal input decay after Cxcr2 stimulation suggests rapid phosphorylation, whereas its full restimulation potency is consistent with recycling. The localization of internalized Cxcr2 close to the plasma membrane is also consistent with a capacity for rapid re-stimulation. This intermittent signaling profile may explain the bidirectional motion pattern elicited by the Cxcr2 and Cxcr1-chim receptors, as this would allow neutrophils to be sequentially stimulated in different parts of the cell, supporting re-polarization and directional changes. Conversely, continuous signaling with little decay between stimulation rounds would not favor bidirectional motion and this is indeed the case for the Cxcr1-ala mutant, which causes focalized motility. It remains unclear why the Cxcr2-ala mutant nevertheless rescued the dispersal defect of *cxcr2*-null mutants. Our evidence indicates that this receptor retains an ability to discriminate sequential signal inputs despite having lost GRK phosphorylation sites, which may suggest receptor-intrinsic factors. Alternatively, the different phenotypes of Cxcr1-ala and Cxcr2-ala neutrophils could reflect differences in the concentration or presentation of the corresponding ligands in vivo. Further studies could explore these unknown determinants of bidirectional motion.

The mechanisms we describe have general relevance in understanding mammalian immune cell trafficking in vivo. Reverse migration has sparked interest since its discovery in zebrafish^6^ and mice^7,8^ due to its implications in resolution of inflammation. Uncovering related mechanisms is envisaged as a potential route for targeting neutrophil inflammation without compromising host defense. Our study shows that neutrophil dispersal post arrival at injury sites requires receptors that can persist at the plasma membrane in the presence of ligand and thereby sustain chemokinesis in the tissue. This is useful in identifying receptors that might execute similar functions in mammals and highlights a possible avenue to manipulate rIM through modulation of receptor trafficking. We consider this insight particularly important, as the functional correspondence of receptors that mediate rIM may not be as translatable from zebrafish to mammals based on orthology, as much as based on receptor biochemistry. For example, our study would suggest mammalian CXCR1 as a better candidate than CXCR2 for functions analogous to zebrafish Cxcr2, as the former is more resistant to internalization than the latter^24^. Beyond chemokines, various chemoattractant receptors are sustained at the plasma membrane, among which the human BLT1 receptor for the attractant LTB4^41^, which, interestingly, has been implicated in rTEM in a pathological mouse model of chronic injury^8^. Beyond neutrophils, sustained chemokinesis is important for random motility of mouse T cells within lymph nodes and initiation of adaptive immune responses^45^. It would be interesting to explore the role of chemokine receptor trafficking in the longevity of chemokinesis in this context, as one of the relevant chemokines (CCL21) does not trigger receptor (CCR7) downregulation^46,47^. Thus, our findings in zebrafish could be extended to various physiological and pathological settings.

In conclusion, we reveal a molecular mechanism that enables appropriate transition from neutrophil congregation to reverse migration and resolution. Similar mechanisms may regulate the migration behavior of other cells, given the broad representation of GPCRs as chemoattractant receptors.

## Methods

### General zebrafish procedures

Zebrafish were maintained in accordance with UK Home Office regulations, UK Animals (Scientific Procedures) Act 1986. At the Wellcome Sanger Institute, zebrafish were maintained under project licence 70/7606, which was reviewed by the Wellcome Trust Sanger Institute Ethical Review Committee. At the University of Cambridge (Department of Physiology, Development, and Neuroscience) zebrafish were maintained under project licence 70/8255, which was reviewed by the University Biomedical Services Committee. Animals were maintained according to ARRIVE guidelines. Zebrafish were bred and maintained under standard conditions at 28.5 ± 0.5 °C on a 14 h light:10 h dark cycle. Embryos were collected from natural spawnings at 4–5 h post fertilization (hpf) and thereafter kept in a temperature-controlled incubator at 28 °C. Embryos were grown at 28 °C in E3 medium, bleached as described in the Zebrafish Book^48^, and then kept in E3 medium supplemented with 0.3 µg/ml of methylene blue and 0.003% 1-phenyl-2-thiourea (Sigma-Aldrich) to prevent melanin synthesis. For live imaging of neutrophils expressing fluorescent receptors, methylene blue was omitted from E3 medium to minimize tissue autofluorescence. All embryos were used between 2.5 and 3.5 dpf, thus before the onset of independent feeding.

### DNA constructs

Complementary DNA for zebrafish Cxcr1 (Entrez ID: 797181; Ensembl gene: ENSDARG00000052088) was obtained by PCR from a zebrafish neutrophil cDNA library. cDNA for zebrafish Cxcr2 (Entrez ID: 796724; Ensembl gene: ENSDARG00000054975) was obtained by PCR from total larval cDNA library. Cxcr1 and Cxcr2 cDNA was fused to a fluorescent timer cassette and cloned into a pCS2^+^ vector for mRNA production or a Tol2 backbone vector carrying a Lysozyme C promoter (Tol2-lyz vector), using KpnI-MfeI sites^30^. Similarly, mCFP was amplified by PCR from the Clontech vector p-ECFP-Mem (catalog number: 6918-1) and cloned into the Tol2-lyz vector using KpnI-MfeI sites. pCMV-Cxcl8a-mCherry was cloned previously by overlapping PCR^35^. Cxcr1-ala-FT mutant and Cxcr1-chim-FT mutant receptors were generated by overlapping PCR. Overlapping primers used for Cxcr1-ala-FT mutant: Fw: 5′-CGCAGAGGTCCCTGCGGCGTTTCTGGACCCAGCTTTCTTGTACAAAGTGG-3′ and Rv: 5′-CAGAAACGCCGCAGGGACCTCTGCGGTCAGAGCAGCTGCCTTGGCGGCTTTAGCAAGCGCGAACCGCTCCAGAACTCCCTT-3′. Overlapping primers used for Cxcr1-chim-FT mutant: Fw: 5′-CGTCGGAGAGAAGTTCAGACGGAGGCGTAACCAGTTGCTGATTTCTCTC-3′ and Rv: 5′-GAGAGAAATCAGCAACTGGTTACGCCTCCGTCTGAACTTCTCTCC-3′. Overlapping primers used for Cxcr2-chim-FT mutant: Fw: 5′-CCATCCTGTACGCCTTCATCGGGAAGAAATTTTTTCTGCAGTTGCTCCACAGGAAGGGAG-3′ and Rv: 5′-CTCCCTTCCTGTGGAGCAACTGCAGAAAAAATTTCTTCCCGATGAAGGCGTACAGGATGG-3′. The Cxcr2-ala mutant was made by synthesis of the segment carrying the mutations (Genewiz) and this segment was cloned into the pCS2^+^-Cxcr2-FT construct using NheI and Bsu36I, and further subcloned into the Tol2-lyz vector using Kpn-Bsu36I. Some of the mutant constructs were cloned into pCS2^+^ vector for mRNA production and all mutant constructs were cloned into the Tol2-Lyz backbone vector for transgenesis. cDNA with the coding sequence for Cxcl8b (NM001327985/XM_003198892) was synthesized by Genewiz and cloned into pCS2^+^ using EcoRI/XhoI sites. This was used for in vitro transcription and HEK293T transfections. A pCS2^+^ plasmid containing Cxcl8a cDNA (XM_001342570)^35^ was used for in vitro transcription and a pCMV vector containing a C-terminal, His-tagged version of Cxcl8a^35^ was used for transfection of HEK29T cells.

### Chemokine receptor internalization assays in early embryos

For mRNA production, the pCS2^+^ plasmids containing the Cxcr1-FT, Cxcr2-FT, Cxcr2-chim-FT, Cxcl8a, and Cxcl8b constructs were linearized and transcribed with an mMessage mMachine SP6 kit (Ambion) followed by polyA addition (Ambion). mCFP template for in vitro transcription was prepared from a Clontech Plasmid containing the cDNA for mCFP (p-ECFP-mem; Catalog #6918-1) and the following primers: Fw: 5′-GGGGATTTAGGTGACACTATAGAAGCCGCCACCATGCTGTGCTGTATGAGA AGAAC-3′, Rv: 5′-TCGCGGCCGCTTTACTTGTACAGCTCGTC-3′. One hundred picograms of Cxcr1/2-FT, mCFP, or Cxcr2-chim-FT, were injected into the cell body of one-cell stage embryos along with 100 pg mECFP mRNA. In some cases, 150 pg Cxcl8a or Cxcl8b mRNA was co-injected. Injected embryos were stored in Petri dishes at 28 °C and imaged at about 8 hpf. For imaging, embryos were de-chorionated using forceps and mounted on glass-bottomed microwell dishes (MatTek) in 0.8% low-melting point agarose covered with 2 ml E3. Embryos were imaged on an inverted Olympus Fluoview FV1000 or Leica SP8 confocal microscope and *z*-stacks were acquired using a ×40/1.25 numerical aperture (NA) silicon objective (Olympus) and ×40/1.3 NA oil objective (Leica). The mCFP, sfGFP, and tagRFP were visualized with 440, 488, and 559 nm excitation wavelengths, respectively, for the Olympus scope and with 405, 488, and 552 nm, respectively, on the Leica scope.

Internalization of receptors in gastrulating embryos was quantified in Fiji. For analysis, a binary membrane mask was made using the cyan channel of the mCFP control by thresholding. This mask was then applied to all three channels (sfGFP, tagRFP, and memECFP) using the image calculator. Ratios of masked images were normalized to the corresponding ratios of the unmasked images. This normalized variation in expression levels across different embryos and allowed pooling of ratios from different samples. The same approach was tested on neutrophils in Supplementary Fig. 1e, f.

### Generation of Cxcr1/2-FT transgenic lines

Solution (1 nl) containing 12.5 ng/µl Tol2 DNA construct and 17.5 ng/µl transposase mRNA (1 nl of 17.5 ng/µl) were injected into the cell body of one-cell stage embryos (either in wild-type AB embryos or *cxcr2*^*−/−*^/*cxcr1*^*−/−*^ embryos for the rescue lines). Transposase mRNA was synthesized from pCS2-TP^49^ by in vitro transcription mMessage mMachine SP6 kit Ambion). Injected embryos were stored at 28 °C until 5 dpf and thereafter were raised in the fish nursery according to standard rearing protocols. At 3 months old, F0 fish were outcrossed to a wild-type (TL) line to screen for germline transgenesis. F1 offspring were kept as separate sub-strains due to differences in expression of the transgenic cassette used. F1 to F3 embryos were used for live imaging.

### Generation and screening of *cxcr1* and *cxcr2* knockout lines

The mutant alleles *cxcr1*^*sa14414*^ (*cxcr1* is otherwise annotated as *si:ch73-54b5*.*2*) and *cxcr2*^*sa6118*^ (hereafter referred to as *cxcr2*^*−/−*^) were generated in the Zebrafish Mutation Project^50^ and subsequently maintained at the Wellcome Trust Sanger Institute through natural matings and in vitro fertilization using frozen sperm as previously described^51^. Heterozygous *cxcr1*^*sa14414*^ (*cxcr1*^*+/−*^) and *cxcr2*^*sa6118*^ (*cxcr2*^*+/−*^) embryos were transferred to University of Cambridge (PDN) for further screening and maintenance. For genotyping, *cxcr1*^*sa14414*^ and *cxcr2*^*sa6118*^ fish were anesthetized in fish water containing 200 µg/ml tricaine and tissue samples were obtained via fin clipping. DNA was extracted using a ThermoScientific Phire Tissue Direct PCR Master Mix or through the protocol described by ref. ^50^. Fish were genotyped using KASP-genotyping chemistry^51^, using two allele-specific primers and a common reverse primer synthesized by LGC. For *cxcr1*^*sa14414*^, primers were as follows: primer_alleleFAM: 5′-AGTGAGAGCACTAAACCCAAAACC-3′, primer_alleleHEX: 5′-CAGTGAGAGCACTAAACCCAAAACT-3′, primer_common: 5′-GTGGAGTCGCKTGCGGATTAGTTT-3′. For *cxcr2*^*sa6118*^ primers were as follows: primer_alleleFAM: 5′-ATCTGATTGGGTTTGTGTGTGCGTT-3′, primer_alleleHEX: 5′-ATCTGATTGGGTTTGTGTGTGCGTA-3′, primer_common: 5′-GGTGCACCATAACCGGAAGAGATAA-3′. KASP genotyping assays were conducted according to the manufacturer’s instructions (https://www.lgcgroup.com/products/kasp-genotyping-chemistry/#.W036CxjMzXQ). Briefly, 100 ng samples of extracted DNA were loaded onto a 384-well PCR plate and left to dry overnight. KASP assays were assembled as follows: 2.5 µl, 0.07 µl primer mix, and 2.43 µl water. Plates were read on a Roche LC480 LightCycler VII and genotypes were assigned to samples using cluster analysis^51^. Fish screened positive for the desired mutation were outcrossed to a Tg(*mpx*:GFP)^*i114*^ transgenic line^52^, to visualize neutrophils during microscopy. Outcrosses were repeated to progressively remove additional mutations carried by the *cxcr1*^*sa14414*^ and *cxcr2*^*sa6118*^ fish. Rescue zebrafish lines were generated using homozygous *cxcr1*^*sa14414*^ *(cxcr1*^*−/−*^*)* screened negative for other mutations identified in the founder fish during the Zebrafish Mutation Project.

### Fin wounds

Wild-type (Tg(*mpx*:GFP)^i114^ ^52^, *cxcr1*^*−/−*^ or *cxcr2*^*−/−*^ larvae and the various rescue lines were anesthetized with 200 µg/ml tricaine (MS222) at 3 dpf and wounded in the ventral fin or tail fin using a sterile surgical scalpel blade (Swann-Morton, 23). A previously validated morpholino targeting the ATG region of cxcr2 (5′-ACTCTGTAGTAGCAGTTTCCATGTT-3′) was injected into the yolk of cxcr1^*−/−*^ embryos (3 nl of 100 µM solution). In some cases, 30 nM of LTB4 (Sigma) was added in E3 for 30 min, transferred to E3 medium (+tricaine), and wounded using a sterile blade as previously described^36^.

### Neutrophil uptake of Cxcl8a

HEK293T cells were cultured in Dulbecco’s modified Eagle’s medium (DMEM) (Invitrogen) containing 10% fetal bovine serum (Gibco ThermoFisher Scientific) and 1% Penicillin/Streptomycin (Sigma). HEK293 cells were transfected with Cxcl8-mcherry using Lipofectamine 2000 (Invitrogen). Transfected cells were incubated at 37 °C (with 5% CO_2_) overnight, collected the following morning, and resuspended in DPBS (Invitrogen) at a density of 30 × 10^6^/ml. Cells were transplanted above the yolk as previously described^35^ into 48hpf Tg(*mpx*::GFP)/wild type or Tg(*mpx*::GFP)/cxcr1^*−/−*^. Transplanted larvae were incubated in E3 medium containing PTU (1-phenyl-2-thiouria) at 34 °C overnight. Eighteen to 24 h post transplantation, larvae were used for imaging (see “live imaging” section) and temperature was maintained at 32–35 °C using a built heated chamber (for the upright scope) or a heated stage (for the inverted scope).

### Chemokine expression analysis and knockdown

Morpholino oligonucleotides (Gene Tools) were injected in the yolk of one-cell-stage embryos. A *cxcl8a* splice-blocking morpholino 5′-ATTTATGCTTACTTGACAATGATC-3′ (12 ng) was used in combination with a *cxcl8a* translation-blocking morpholino 5′-TTTGCTGGTCATTTTGCCTAAGTGA-3′ (9 ng)^35^. A *cxcl8b* splice-blocking morpholino was used alone 5′-TTAGTATCTGCTTACCCTCATTGGC-3′ (4 ng)^33^. For genotyping the splice-blocking morpholinos, RNA was prepared from pools of ten injected or non-injected 3 dpf larvae using the RNeasy Mini Kit (Qiagen). cDNA was prepared using M-MLV reverse transcriptase (Invitrogen) and used for RT-PCR. The following primers were used: *cxcl8a* forward: 5′-GCCACCTTGATGACAACTGGA-3′, *cxcl8a* reverse: 5′-TGTCTGACGTATGAACATCATCAAAC-3′; *cxcl8b* forward: 5′-GATGAAGTTGAGCGTTTCAGCC-3′, *cxcl8b* reverse: 5′-GAAATCACCCACGTCTCGGTAG-3′. The following primers were used for the housekeeping gene: *ef1a* forward: 5′-GCTGATCGTTGGAGTCAACA-3′, *ef1a* reverse: 5′-ACAGACTTGACCTCAGTGGT-3′. For comparison of *cxcl8a* and *cxcl8b* expression levels in wounded vs. unwounded larvae, 3 dpf larvae were wounded in the ventral fin and RNA was extracted from pools of ten larvae at 30 mpw, 1.5 hpw and 2 hpw. The following primers were used for RT-PCR: *cxcl8a* forward: 5′-ATGAGCTTGAGAGGTCTGGC-3′, *cxcl8a* reverse: 5′-GTGATCCGGGCATTCATGG-3′ (for cxcl8b, the same primers as for morphotyping were used).

### In vitro chemotaxis assays

Transwell chemotaxis assays were performed as previously described^35^. Briefly, whole kidney marrow was extracted from Tg(*mpx*:GFP), Tg(*mpx*:GFP)/*cxcr1*^*−/−*^, or Tg(*mpx*:GFP)/*cxcr2*^*−/−*^. Single-cell suspensions were made and cells were placed at a density of 100,000 cells/well in the top chamber of a 96-well HTS Transwell system with 3 μm pore-size polycarbonate membrane filters (Corning). Recombinant Cxcl8a was added to the bottom wells at the indicated concentrations. After incubation for 3 h at 28 °C, cells in the bottom chamber were collected and the number of GFP^+^ cells was assessed by fluorescence-activated cell sorting (FACS) analysis (FACScan, BD) on a Cytek DxP8. A 20 mW 488 nm laser was used to excite GFP and a 530/30 bandpass filter for detection. Cells and beads were acquired using a 530/30-H vs. SSC-H and at least 5000 events were recorded per sample. The analysis was done on FlowJo 10.4.1.The results were normalized to Calibrite beads (BD), which had been added to each cell sample just before collection, to ensure independence from volume and FACS fluctuations.

### Live imaging of zebrafish neutrophils

Fish larvae were mounted immediately after wounding (or 18–24 h post transplantation) onto a glass-bottom plate in 1% low-melting agarose (Invitrogen) or a custom-built coverslip chamber (for when using an upright scope). Agarose-embedded embryos were covered with 2 ml E3 medium (supplemented with tricaine) and imaged either on (i) an inverted PerkinElmer UltraVIEW ERS, Olympus IX81 spinning disk confocal microscope with a ×20 /0.45 NA air objective (Olympus) or ×30/1.05 NA silicon (Olympus), or ×40/1.25 NA silicon objective (Olympus) and 405 nm for CFP excitation, 488 nm for GFP excitation, and 561 for tagRFP or mCherry, or (ii) on an upright Nikon E1000 microscope coupled to a Yokogawa CSU10 spinning disc confocal scanner unit with a ×20/0.75 NA air objective (Nikon) or ×10/0.5 NA air objective (Nikon) and illuminated using a Spectral Applied Research LMM5 laser module (491 nm for GFP excitation; 561 nm for Ruby or TagRFP or mCherry). Confocal stacks using a 2 µm *z*-spacing were acquired every 30 s.

### Sudan black staining

Tg(*mpx*::GFP)/*wildtype*, Tg(*mpx*::GFP)/*cxcr1*^*−/−*^, and Tg(*mpx*::GFP)/*cxcr2*^*−/−*^ 3 dpf larvae were wounded in the ventral fin with a scalpel blade and fixed 20–24 h later in 1 ml of 4% ethanol-free formaldehyde (ThermoScientific) in phosphate-buffered saline (PBS; Sigma-Aldrich) overnight at 4 °C with agitation. Fixed larvae were rinsed in PBT (PBS with 0.1% Tween-20; Sigma-Aldrich) twice for 5 min and incubated in 1 ml Sudan Black (Sigma-Aldrich) for 15 min. Following staining, larvae were washed in 70% ethanol for several hours and passaged into 30% ethanol overnight at 4 °C with agitation. Larvae were washed in PBT for ten minutes, passaged into increasing concentrations of glycerol and stored in 80% glycerol at 4 °C.

### Cell culture

Tissue culture media, reagents, and PTX were purchased from ThermoFisher Scientific (Karlsruhe, Germany). Recombinant Cxcl8a was produced by Proteintech using a bacterial expression system (Manchester, UK). The produce sequence contained two additional N-terminal amino acids and a C-terminal His-tag (sequence produced: (MGMSLRGLAVDPRCRCIETESRRIGKHIKSVELFPPSPHCKDLEIIATLMTTGQEICLDPSAPWVKKIIDRIIVNRKPLEHHHHHH). The FR900359 used in all experiments was isolated from the dried leaves of *Ardisia crenata* as previously described^40^. Cells were cultured at 37 °C in a humidified atmosphere of 95% air and 5% CO_2_. HEK293T cells were obtained from the American Type Culture Collection and parental HEK293 were from ThermoFisher Scientific (Karlsruhe, Germany). HEK293T and parental HEK293 were cultivated in DMEM supplemented with 10% (v/v) fetal calf serum (FCS), the antibiotics penicillin (100 U/ml) and streptomycin (100 µg/ml). Cell lines expressing zebrafish Cxcr1 and Cxcr2 receptors were generated by stable transfection of parental HEK293 and subsequent selection with G418 (500 µg/ml, InvivoGen, Toulouse, France). For preparation of Cxcl8a/Cxcl8b supernatant, HEK293T cells were transfected with the corresponding constructs (see “DNA constructs” section) using polyethylenimine. Six hours later, medium was replaced with culture medium without FCS. After 18 h conditioned medium containing secreted Cxcl8b was collected and stored at −20 °C for use in DMR assays and studies of receptor internalization.

### Label-free DMR assays

HEK293T cells were transfected with receptor (800 ng in 50 µl Opti-MEM) using Lipofectamine 2000 (ThermoFisher Scientific) according to the manufacturer’s instructions. In brief, plasmid DNA was mixed with Lipofectamine 2000 (2 μl in 50 μl Opti-MEM) and incubated for 5 min at room temperature. The transfection mixture (100 μl) was added to the freshly trypsinized HEK293T cells (600,000 cells in 900 µl complete media) and seeded in 384-well fibronectin-coated EPIC biosensor plates (Corning; 15,000 cells/well). On the next day, PTX (100 ng/ml) was added. At 48 h post transfection, real-time whole-cell DMR measurements were conducted as previously described in detail^37,38^. Briefly, cells were washed twice with Hank’s Balanced Salt Solution (HSBB) containing 20 mM HEPES (pH 7.2) and 0.5% fatty acid-free bovine serum albumin (BSA) (Sigma-Aldrich, Steinheim, Germany) and incubated for 1 h at 37 °C in the EPIC benchtop reader (Corning) for temperature equilibration. During this pre-incubation time, FR900359 was added at a final concentration of 1 µM. The sensor plate was scanned for a baseline optical read of about 3 min and then agonist was added with a semi-automated liquid handling robotic (Selma, CyBio^®^) and DMR changes were recorded for 1 h at 37 °C. In restimulation experiments using parental HEK293 cells stably expressing chemokine receptors, second ligand addition was performed 30 min after the first stimulation. All real-time recordings show one representative biological replicate as mean + SEM of three technical replicates. Quantification of DMR signals was performed by calculation of the maximum responses or areas under curve as indicated.

### Imaging of HEK293T cells

Microscopy was carried out on an AxioObserver inverted fluorescence microscope (Zeiss). For live-cell imaging of FT-tagged Cxcr1 and Cxcr2, stable cells were seeded onto fibronectin-coated 8-well µ-Slides (Ibidi) and cultured overnight at 37 °C and 5% CO_2_. The next day, cells were washed three times with HBSS containing 20 mM HEPES and 0.5% fatty acid-free BSA, and stimulated either with buffer, recombinant Cxcl8a, or Cxcl8b supernatant at 37 °C for 15 and 30 min. Fluorescence images were obtained using an ApoTome Imaging System with a Plan-Apochromat ×63/1.40 Oil DIC and the filter set 38 (green).

### Statistics

All error bars indicate SEM. All *p*-values were calculated with two-tailed statistical tests and 95% confidence intervals. A *t*-test (pairwise comparisons) and one-way analysis of variance (multiple group comparisons) with Dunnet’s post test were performed after distribution was tested for normality, otherwise non-parametric tests were performed (Mann–Whitney for two-way comparisons and Kruskal–Wallis with Dunn’s post test for multiple comparisons). Statistical tests were performed in Prism v8.0.2 (GraphPad Software, Inc., La Jolla, CA). The statistical test and the *n* number are indicated in the figure legends. The error bars show SEM either across individual embryos (i.e., analysis of neutrophil recruitment, cluster size, and net reverse traffic) or individual neutrophils pooled from different embryos (i.e., track straightness, which is a track-based analysis, or speed vs. distance or orientation, which are step-based analyses). Live-imaging experiments were acquired in minimum two independent imaging sessions unless otherwise indicated.

### Extraction and classification of neutrophil trajectories

Analysis of neutrophil trajectories was performed in Imaris v8.2 (Bitplane AG, Zürich, Switzerland) on a two-dimensional (2D) maximum intensity projections of the four-dimensional time-lapse movies. Unless otherwise indicated, analyzed trajectories were extracted from the whole fin area to account for interstitial movement (movement in the CHT was excluded). A track duration threshold of three time points was defined to exclude short-lived tracks. Manual track corrections were also applied where needed. Instantaneous neutrophil coordinates over time (*x, y, t*) were exported into Microsoft Excel 2016 spreadsheet files (Microsoft Corporation, Redmond, WA).

Exported Excel files were imported into MATLAB R2018b (The MathWorks, Inc., Natick, MA) using a custom-written script, for neutrophil trajectory analysis. For each experimental image dataset, the area of the wound occupied by the neutrophil cluster (owa) was defined by a set of manually selected points (*x*, *y*). To define the owa, we used a maximum intensity time projection of the movies, in which high-density neutrophil areas could be distinguished based on intensity levels. Separation of trajectories into forward and reverse segments was done as follows: for forward trajectory segments, the first time point of the trajectory segment was defined as the first time point the neutrophil was detected in the fin and the last time point of the trajectory was defined as the time point before entering the owa. For reverse trajectory segments, the first time point was defined as the last time point the neutrophil was detected within the owa, whereas the last time point was taken as the last time point it was detected in the fin. In speed–distance and speed–cosine plots, forward tracks also included tracks that did not intersect with the owa, but whose direction of movement could be defined as forward, based on the end position of the track being located closer to the owa than the start position. In the “net reverse traffic” plots, cells not intersecting the owa were not included, to exclude contribution of tracks that did not pass by the owa. For track straightness plots, no classification on forward/reverse tracks was performed. Data were binned using custom-written scripts.

### Analysis of speed vs. distance/orientation from the owa

For analysis of speed vs. distance from the owa, we used custom MATLAB scripts modified from previously used scripts^35^. The instantaneous speed was calculated based on the distance traveled by a neutrophil between two successive time points The distance from owa was defined as the distance between the position of a neutrophil centroid (*x*, *y* coordinates) and the nearest point of the owa perimeter.

For analysis of speed vs. orientation in relation to the owa, we calculated the instantaneous speed as above. The angle *θ* was calculated as the angle between the vector of the neutrophil instantaneous speed and the vector that connects the neutrophil initial position with the nearest point in the owa perimeter (Fig. 3d). The migration orientation effect was calculated using the cosine of angle *θ* within a range of 50 μm from the owa perimeter. A value of cosine *θ* closer to +1 shows directed migration towards the wound, whereas a value closer to −1 shows migration away from the wound.

### Calculation of track straightness

The track straightness was calculated as the distance that a neutrophil traveled between the first and last time point of its trajectory, divided by the cumulative distance traveled in the same time window, using a MATLAB custom-written script. A track straightness value closer to 1 showed a direct migration, while a track straightness value closer to 0 showed an arbitrary motion. For each track, data from neutrophil coordinates corresponding to positions inside a range of 50 µm were included. Tracks of total length of <10 μm were eliminated to exclude neutrophils that did not show sufficient movement to calculate representative straightness. Data were binned using custom-written scripts.

### Calculation of net reverse traffic

The net traffic was calculated as the number of reverse neutrophil tracks, divided by the number of forward migrating neutrophil tracks. The higher the value, the higher the net reverse traffic.

### Receptor internalization analysis

Analysis of Cxcr1 or Cxcr2 receptor internalization was performed in MATLAB using custom-written scripts. Neutrophil or cluster outline definition was achieved with active contours using MATLAB’s built-in function for the Chan-Vese method^53^. To define the core of the neutrophil, a 2D point *(x*, *y)* inside each neutrophil was selected manually and expanded for 5–15 pixels in each direction (−*x*, +*x*, −*y*, +*y*). The active contour algorithm expanded this core until the neutrophil boundary. For surface segmentation, all pixels inside the contour were included to define a binary surface mask that was applied on the sfGFP channel. In this segmentation process, a threshold on the size of objects (50 pixels) was applied to eliminate small false detected objects. For contour-based membrane segmentation (Supplementary Fig. 1), the pixels comprising the outline of each neutrophil or cluster were used to define a binary membrane mask that was applied on the mCFP and sfGFP channels. Segmentation was carried out on a representative snapshot of neutrophil migration at around 1–1.5 hpw, indicating the overall internalization level after neutrophils clustered. Only cells that were entirely visible (and not partially) were segmented. Overly dim (not enough signal) or overly bright cells (saturated) with unreliable intracellular signal distribution were not segmented for these analyses.

For datasets in Fig. 2f, for each embryo, the overall quantification had some differences to increase the number of datapoints for profiling the gradient. Data from all time points between 15 and 45 mpw were included. For this reason, the segmentation was more crudely (not on selected cells) using an intensity threshold on the entire image.

Neutrophil contrast, reflecting intracellular heterogeneity in signal distribution, was calculated in segmented neutrophils, using MATLAB’s built-in functions. The intensity matrix of each surface-segmented neutrophil was transformed into a gray-level co-occurrence matrix^32^. The latter represents the intensity difference between neighboring pixels. Contrast was calculated as the difference in intensity relationships, based on the following equation (https://uk.mathworks.com/help/images/ref/graycoprops.html):

$$C = {\int}_{i,j = 0}^{N - 1} {\left| {i - j} \right|^2p(i,j)}$$

where *p(i, j)* is the image co-occurrence matrix and *i*, *j* are the co-occurrence matrix coordinates.

For datasets in Figs. 2d and 4, and Supplementary Fig. 11, contrast values of neutrophils at the wound were normalized to the contrast values of neutrophils in the CHT of the same time point to account for image intensity fluctuations across embryos acquired with independent imaging settings. For datasets in Fig. 2f, normalization with the contrast of cells in the CHT was not applicable as neutrophils were induced to exit the CHT prior to imaging. In this case, the contrast of neutrophils was normalized to the maximum contrast value per individual embryo. For plotting the contrast over distance in Fig. 2f, the distance of the neutrophil centroid from the nearest point of the wound margin was calculated. For calculating the ratio of GFP/CFP in contour-based segmented neutrophils, a ratiometric GFP/CFP image was generated in MATLAB and the mean intensity of each neutrophil was computed from this image.

### Quantification of Cxcl8a chemokine uptake

For calculation of Cxcl8a uptake, GFP-positive neutrophils were segmented based on active contours, as described above. The resulting GFP binary mask was applied on the mCherry images to obtain the intracellular mCherry signal. Neutrophil mean intensity was calculated per cell and normalized with the mean intensity of a 150 × 150 pixels of the transplant. This was to account for variation in levels of mCherry expression in the transplanted cells across independent embryos.

### Calculation of neutrophil cluster size

Neutrophil cluster size at wound was calculated in Imaris. Neutrophils were tracked as a surface, rather than as neutrophil centroids, within a square area approximating the owa. The area of segmented objects (neutrophils) was computed in Imaris and imported in MATLAB for plotting. Surfaces with area below 60 µm^2^ were excluded to minimize artefacts from erroneous surface detection. Cluster size per embryo was calculated as an average across an indicated time window.

### Reporting summary

Further information on research design is available in the Nature Research Reporting Summary linked to this article.

## Supplementary information

Supplementary Information

Reporting Summary

Description of Additional Supplementary Files

Supplementary Movie 1

Supplementary Movie 2

Supplementary Movie 3

Supplementary Movie 4

Supplementary Movie 5

Supplementary Movie 6

Supplementary Movie 7

Supplementary Movie 8

Supplementary Movie 9

Supplementary Movie 10

Supplementary Movie 11

Supplementary Movie 12

Supplementary Movie 13

## Data Availability

The data supporting the findings of this study are available within the Article, Supplementary Information files, and Source data. Source data for the figures are included in the Source Data file. Additional data are available upon reasonable requests. Genetic constructs generated in this study are available from the corresponding author upon request. Genetically modified animals generated in this study are available from the corresponding author upon request, subject to institutional and ethical approvals.

## Source data

Source Data

## Code availability

The Matlab codes and functions can be found in GitHub at https://github.com/LeukocyteMotionAndDynamics/ReceptorTraffic
