# Author Correction: Chemokine receptor trafficking coordinates neutrophil clustering and dispersal at wounds in zebrafish

**DOI:** 10.1038/s41467-019-14041-0

**Published:** 2020-01-21

**Authors:** Caroline Coombs, Antonios Georgantzoglou, Hazel A. Walker, Julian Patt, Nicole Merten, Hugo Poplimont, Elisabeth M. Busch-Nentwich, Sarah Williams, Christina Kotsi, Evi Kostenis, Milka Sarris

**Affiliations:** 1 Department of Physiology, Development and Neuroscience, University of Cambridge, Downing Site, Cambridge, CB2 3DY UK; 2 Institute for Pharmaceutical Biology, University of Bonn, Nussallee 6, 53115 Bonn, Germany; 3 Wellcome Sanger Institute, Wellcome Genome Campus, Hinxton, CB10 1SA UK; 4 Department of Medicine, University of Cambridge, Cambridge, CB2 0QQ UK

**Keywords:** Amoeboid migration, Chemotaxis, Imaging the immune system, Neutrophils

Correction to: *Nature Communications* 10.1038/s41467-019-13107-3, published online 14 November 2019.

The original version of this Article contained an error in the Supplementary Information file, in which Supplementary Fig. 7 was inadvertently reproduced to overwrite Supplementary Fig. 12, resulting in the absence of intended data. The HTML has been updated to include a corrected version of the Supplementary Information.

