# Supplementary material for: Chemokine receptor trafficking coordinates neutrophil clustering and dispersal at wounds in zebrafish
Source: Nat Commun. 2019 Nov 14;10:5166. doi: 10.1038/s41467-019-13107-3 (PMC6856356; doi:10.1038/s41467-019-13107-3)
Supplement: Supplementary file 1 — Supplementary Information [file 41467_2019_13107_MOESM1_ESM.pdf]

Chemokine receptor trafficking coordinates neutrophil clustering and dispersal at wounds in zebrafish

Coombs et al.

Supplementary Information

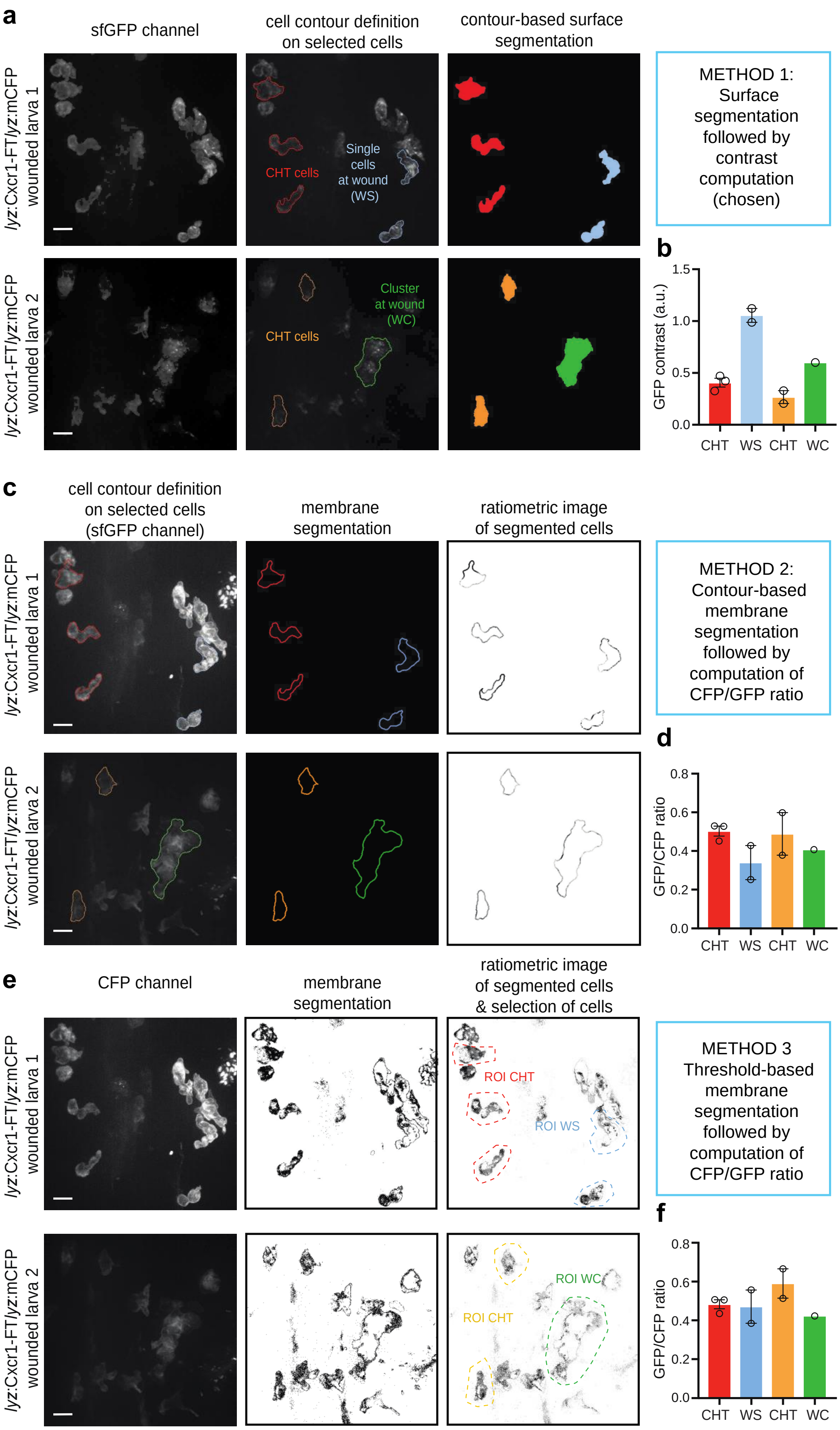
a
sfGFP channel
cell contour definition on selected cells
contour-based surface segmentation
lyz:Cxcr1-FT/lyz:mCFP wounded larva 1
CHT cells
Single cells at wound (WS)
lyz:Cxcr1-FT/lyz:mCFP wounded larva 2
Cluster at wound (WC)
CHT cells
METHOD 1: Surface segmentation followed by contrast computation (chosen)
b
GFP contrast (a.u.)
1.5
1.0
0.5
0.0
CHT WS CHT WC
c
cell contour definition on selected cells (sfGFP channel)
membrane segmentation
ratiometric image of segmented cells
lyz:Cxcr1-FT/lyz:mCFP wounded larva 1
lyz:Cxcr1-FT/lyz:mCFP wounded larva 2
METHOD 2: Contour-based membrane segmentation followed by computation of CFP/GFP ratio
d
GFP/CFP ratio
0.8
0.6
0.4
0.2
0.0
CHT WS CHT WC
e
CFP channel
membrane segmentation
ratiometric image of segmented cells & selection of cells
lyz:Cxcr1-FT/lyz:mCFP wounded larva 1
ROI CHT
ROI WS
lyz:Cxcr1-FT/lyz:mCFP wounded larva 2
ROI WC
ROI CHT
METHOD 3 Threshold-based membrane segmentation followed by computation of CFP/GFP ratio
f
GFP/CFP ratio
0.8
0.6
0.4
0.2
0.0
CHT WS CHT WC

Figure 1. Comparison of quantification approaches for receptor internalization. Single (blue) or clustered neutrophils (green) at wounds or non-mobilized neutrophils in the CHT (red, orange) were segmented and analyzed by different methods to compare results. The same example cells are shown analyzed with three methods. a) The surface of the selected, example cells was segmented based on contour definition in the sfGFP channel. b) Contrast was computed from the example cells shown in a. c) The membrane of the selected, example cells was segmented based on contour definition in the CFP channel. Ratiometric analysis of sfGFP/CFP followed. d) The ratio of sfGFP/CFP was computed on the example cells shown in c. e) The membrane was segmented globally in the whole image using intensity thresholding on the CFP channel. The same cells as in a and c were subsequently selected as regions of interest in a ratiometric image to calculate the sfGFP/CFP ratio. f) The ratio of sfGFP/CFP was computed on the example cells shown in e. Error bars represent S.E.M. from individual cells, in cases of n>1. Scale bar = 10 µm. Source data are provided as a Source Data file.

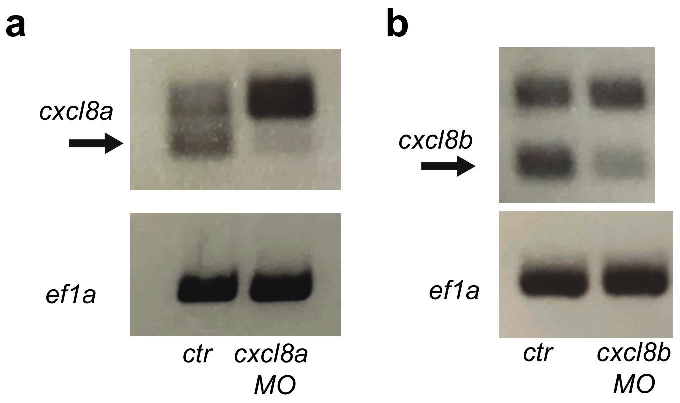

Figure 2. Validation of knockdown of *cxcl8a* and *cxcl8b* expression.

a,b) RT-PCR results showing detection of *cxcl8a* (a) and *cxcl8b* (b) levels in the presence or absence of corresponding splice-blocking morpholino (MO) treatment in 3 dpf larvae. Results from *ef1a* expression detection are shown for comparison. Specific band (150 bp for *cxcl8a*, 254 bp for *cxcl8b* and 435 bp for *ef1a*) is shown with arrows where applicable. Source data are provided as a Source Data file.

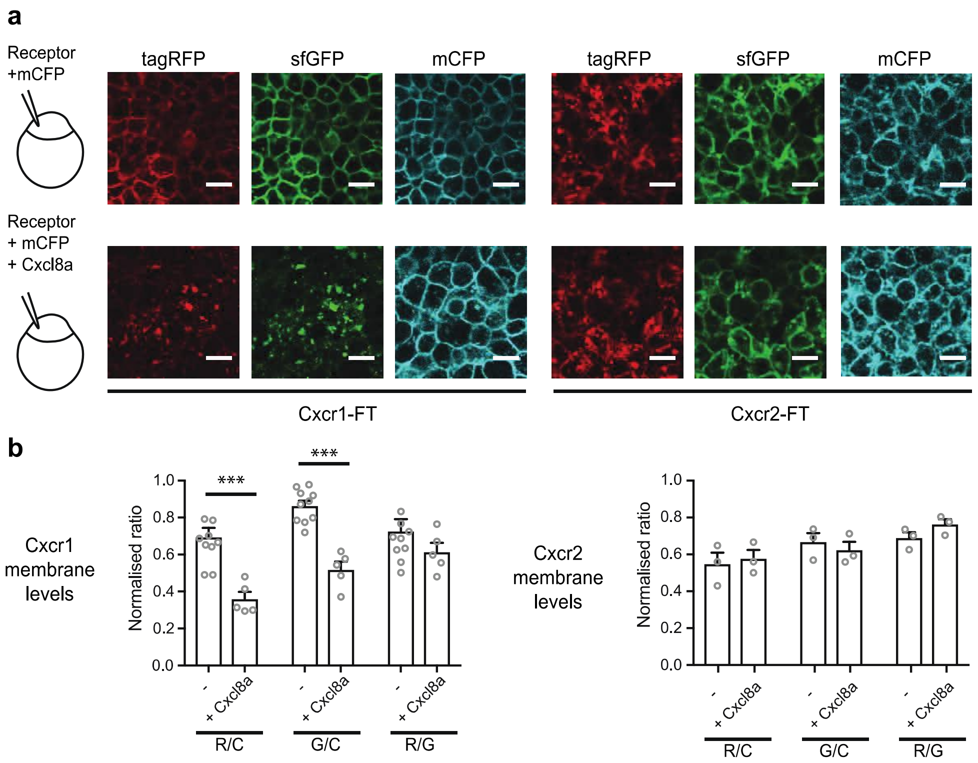

Figure 3. Cxcr1 internalizes in response to Cxcl8a binding.

a) 100pg of Cxcr1-FT or Cxcr2-FT mRNA was injected into one cell-stage eggs with or without 150pg Cxcl8a mRNA. Laser-scanning confocal slices of gastrulating embryos showing expression and distribution of Cxcr1-FT and Cxcr2-FT. Green and red receptors are shown in separate channels. Control membrane marker (mCFP) is shown in the cyan channel. Scale bar = 20 µm b) Quantification of Cxcr1 and Cxcr2 membrane expression as a function of mCFP. Ratio of red over cyan fluorescence (R/C), green over cyan (G/C) and red over green fluorescence (R/G) at the cell membrane is shown. Mean R/C, G/C or R/G ratios at the cell membrane were calculated from ratiometric images after global membrane segmentation (membrane ratio). The membrane ratios were normalized to mean R/C, G/C or R/G ratios of non-segmented ratiometric images of the corresponding source samples. n=10 embryos for Cxcr1 from 4 imaging sessions, n=6 embryos for Cxcr1+Cxcl8a from 3 imaging sessions, n=3 embryos for Cxcr2 and n=3 embryos for Cxcr2+Cxcl8a from 1 imaging

session. Two-tailed Mann-Whitney test. Error bars represent S.E.M. from individual embryos. Source data are provided as a Source Data file.

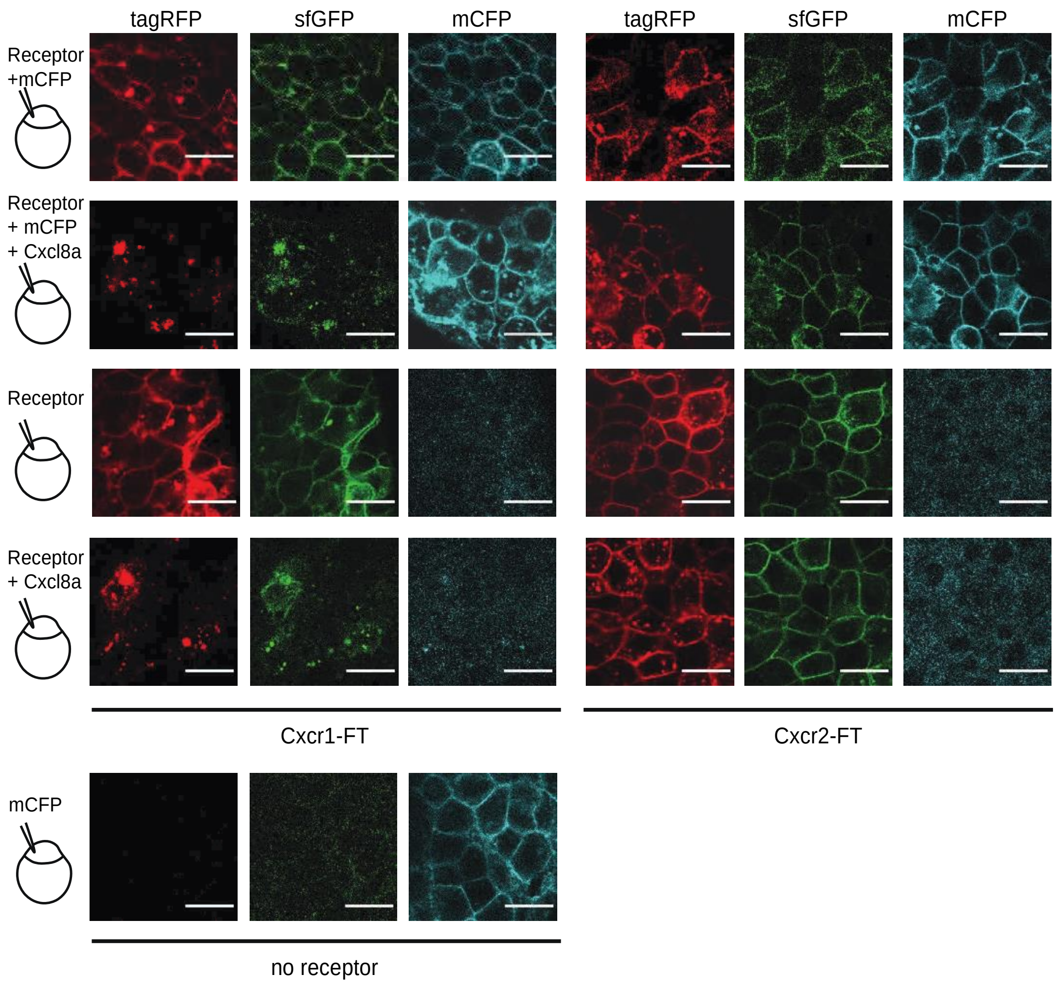

Figure 4. GFP/CFP crosstalk does not account for Cxcr1 and Cxcr2 differences in trafficking in response to Cxcl8a.

100pg of Cxcr1-FT or Cxcr2-FT mRNA was injected into one-cell stage eggs with or without 150pg Cxcl8a mRNA and with or without mCFP. Laser-scanning confocal slices of gastrulating embryos showing expression and distribution of Cxcr1-FT and Cxcr2-FT. Green and red receptors are shown in separate channels. Control membrane marker is shown in the cyan channel. Images were acquired with the same imaging settings to evaluate crosstalk between channels. Scale bar = 20 µm.

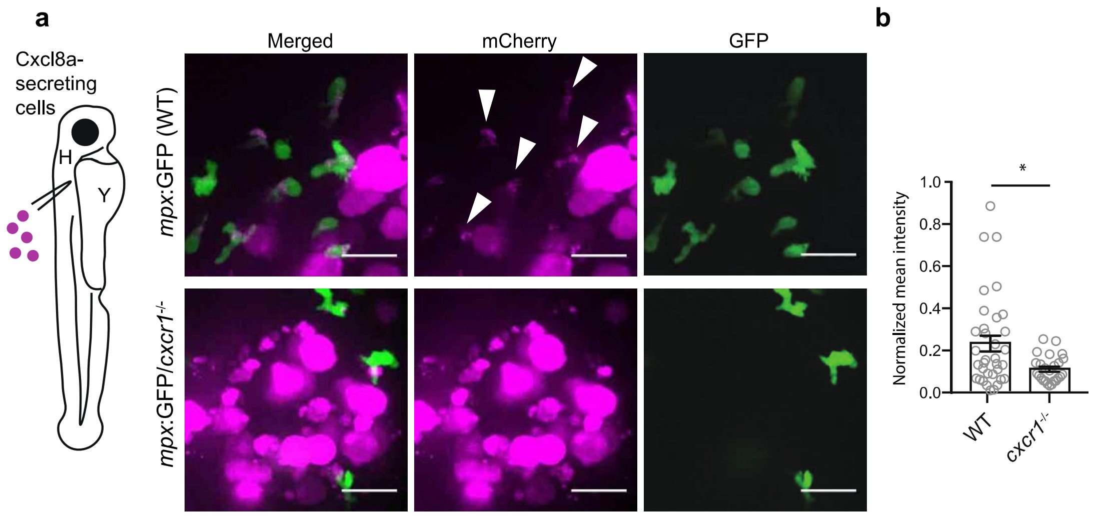

Figure 5. Cxcl8a internalization is Cxcr1-dependent in neutrophils.

a) Cartoon of larva indicating the area of transplantation of Cxcl8a-secreting HEK293T cells. H: head; Y: yolk sac. Confocal projections of Tg(*mpx*:GFP)^{i114} neutrophils, in wild type or *cxcr1*$^{-/-}$ background, responding to Cxcl8a-mCherry-secreting HEK293T cells. Arrows point to internalized Cxcl8a-mCherry in GFP^{+} neutrophils. Scale bar = 25 µm. b) Normalized neutrophil mean intensity. n=34 for Tg(*mpx*:GFP)^{i114} and n=25 cells for Tg(*mpx*:GFP)^{i114}/*cxcr1*$^{-/-}$ from 3 independent larvae per condition from 2 and 1 imaging sessions respectively. Error bars represent standard error of the mean. Two-tailed Mann-Whitney test. Source data are provided as a Source Data file.

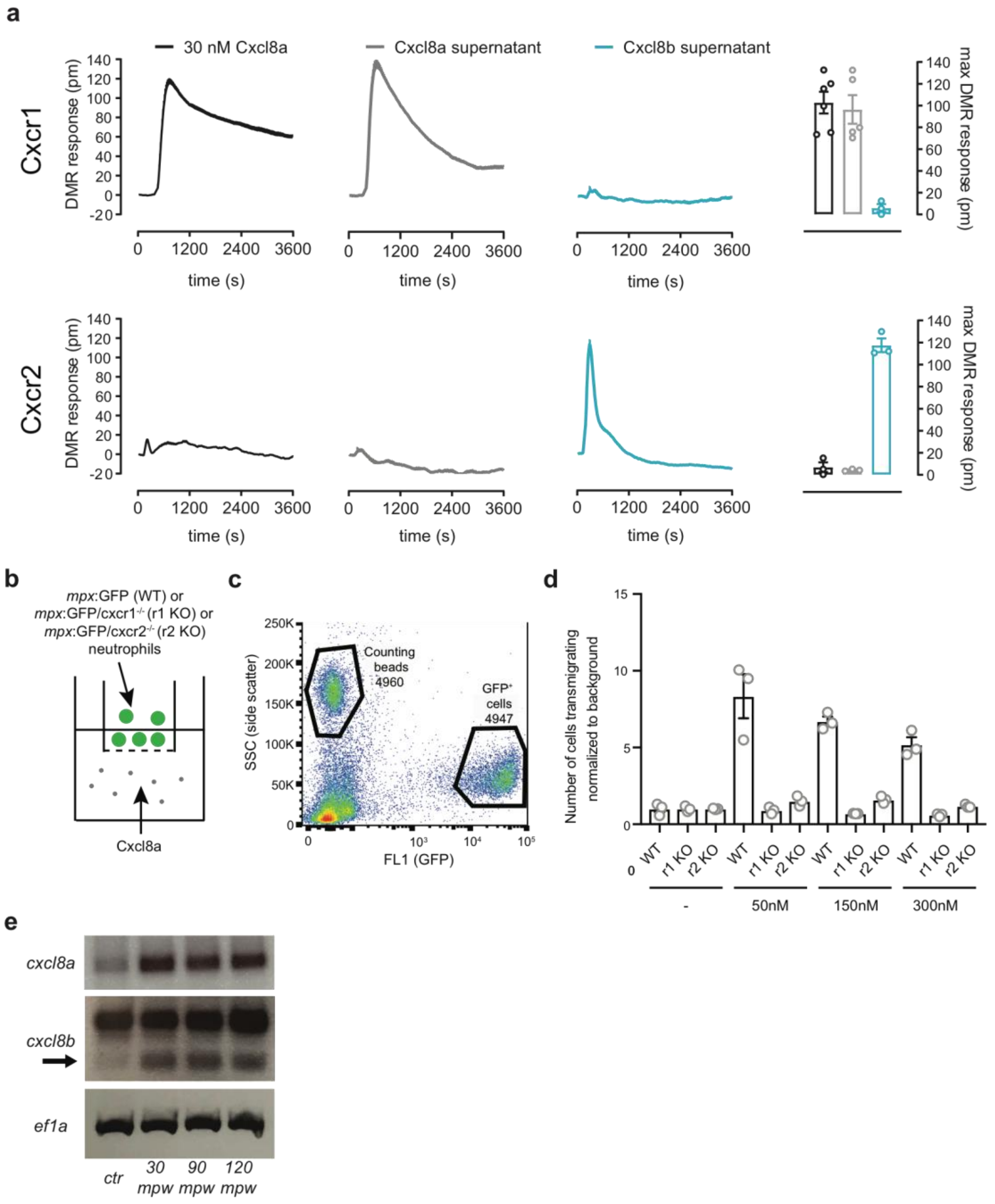

Figure 6. Cxcr1 and Cxcr2 recognize Cxcl8a and Cxcl8b respectively but both genetically contribute to Cxcl8a responses.

a) Real-time DMR recordings of whole cell activity induced by Cxcl8a or Cxcl8b in HEK293 cells transiently or stably expressing Cxcr1 or Cxcr2. DMR recordings are representative for each condition and depict the mean + S.E.M. of a technical triplicate. Error bars in bar graphs represent S.E.M. of 3-6 independent biological replicates. b) Schematic of transwell chemotaxis assay (left). Neutrophils from

Tg(*mpx*:GFP)^{i114} larvae in wild type (WT), *cxcr1*$^{-/-}$ (r1 KO) or *cxcr2*$^{-/-}$ background (r2 KO) were placed in the top chamber and RPMI medium with the indicated concentrations of Cxcl8a was placed in the bottom chamber. c) FACS analysis of cells harvested from the lower chamber. Gating scheme used for GFP^{+} neutrophils and counting beads. Number of cells within gates is shown. (d) Representative results from one chemotaxis experiment out of three. The number of neutrophils in the bottom chamber in various conditions was normalized to the same number of counting beads and to background levels of neutrophil migration towards medium. Error bar indicates S.E.M. from triplicate wells in the assay. e) RT-PCR results from 3 dpf unwounded larvae versus wounded larvae at the indicated times post wound. mpw = minutes post wound. Specific band (244 bp for *cxcl8a*, 254 bp for *cxcl8b* and 435 bp for *ef1a*) is shown with arrows where applicable. Source data are provided as a Source Data file.

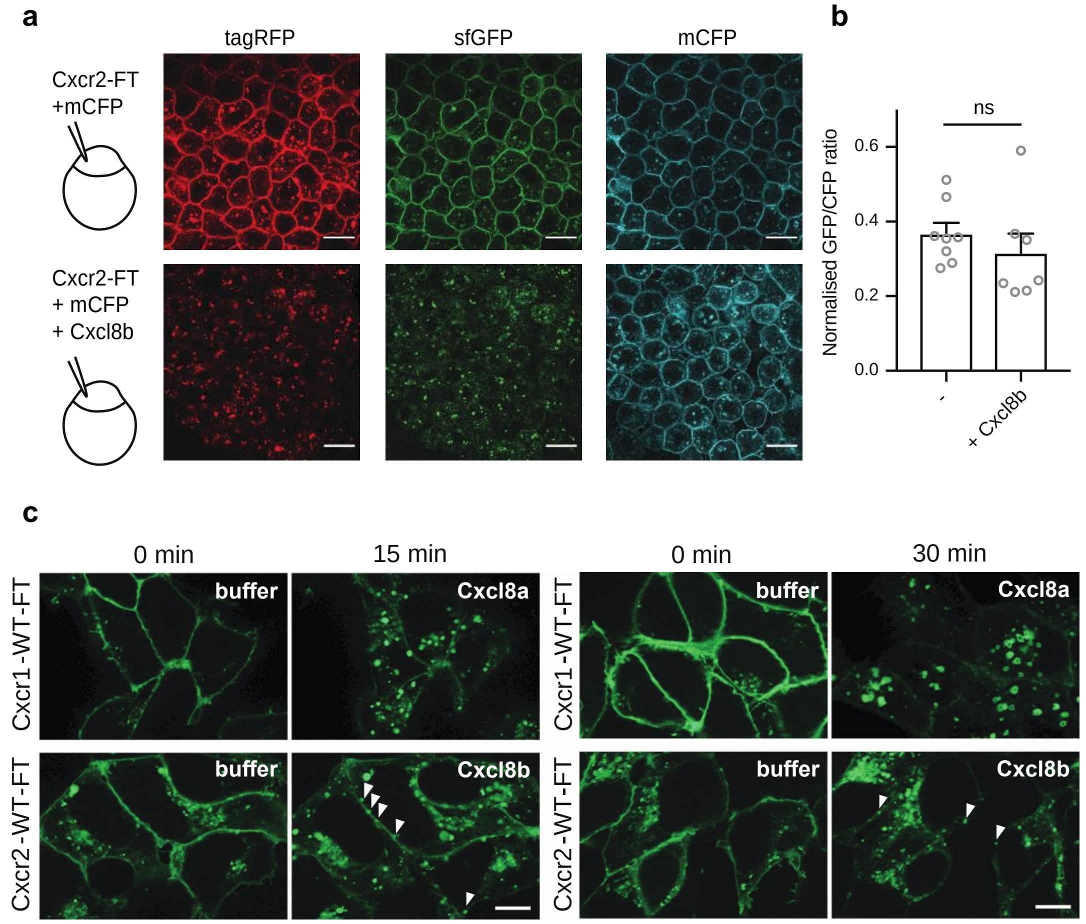

Figure 7. **Comparison of trafficking patterns of Cxcr1 and Cxcr2.**

a) 100pg of Cxcr2-FT mRNA was injected with or without 150pg of Cxcl8b mRNA in one-cell stage embryos. Laser-scanning confocal slices of gastrulating embryos show expression and distribution of Cxcr2-FT. Green and red receptors are shown in separate channels. Control membrane marker (mCFP) is shown in the cyan channel. Scale bar = 20 µm. b) Quantification of wild type Cxcr2 (Cxcr2-WT) membrane expression as a function of mCFP. Mean ratio of GFP/CFP fluorescence at the cell membrane was calculated from ratiometric images after global membrane segmentation (membrane ratio). The membrane ratio was normalized to the mean GFP/CFP ratio of non-segmented ratiometric images of the corresponding source samples. n=8 embryos for Cxcr2 from 2 imaging sessions, n=7 Cxcr2 + Cxcl8b embryos from 2 imaging sessions. Two-tailed Mann-Whitney test. Error bars

represent S.E.M. from individual embryos. c) Representative live-cell fluorescence microscopy images collected in HEK293 cells showing cellular relocation of Cxcr1-FT and Cxcr2-FT in response to their activating ligands Cxcl8a (80 nM) and Cxcl8b (supernatant, 1:4 dilution), respectively. One out of three independent experiments is shown. Scale bar = 10 µm. Source data are provided as a Source Data file.

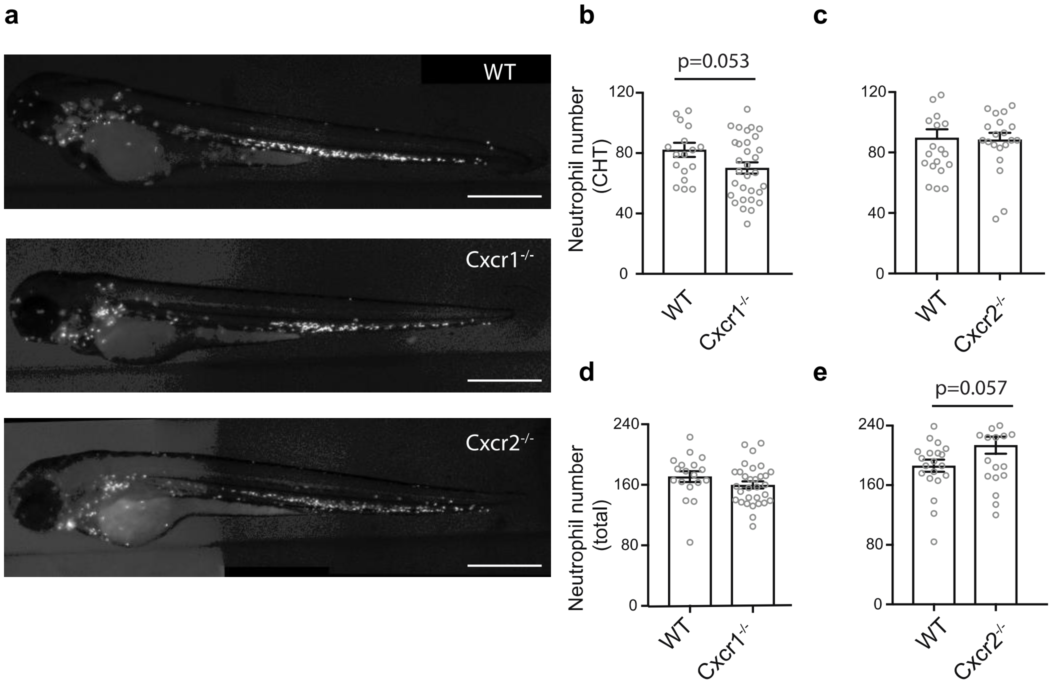

Figure 8. Neutrophil distribution and numbers in wild type, *cxcr1*$^{-/-}$ or *cxcr2*$^{-/-}$ Tg(*mpx*:GFP)^{i114} larvae.

a) Fluorescent microscopy images showing overall neutrophil distribution in non-challenged 3 dpf (b,d) and 3-3.5 dpf (c,e) larvae in wild type, *cxcr1*$^{-/-}$ or *cxcr2*$^{-/-}$ larvae in a Tg(*mpx*:GFP)^{i114} background. Scale bar = 500 µm. b,c) Neutrophil counts in the CHT of *cxcr1*$^{-/-}$/Tg(*mpx*:GFP)^{i114} and *cxcr2*$^{-/-}$/Tg(*mpx*:GFP)^{i114} fish versus wild type. c,e) Neutrophil total embryo count in *cxcr1*$^{-/-}$/Tg(*mpx*:GFP)^{i114} and *cxcr2*$^{-/-}$/Tg(*mpx*:GFP)^{i114} fish versus wild type. (b-e). n=18 wild type and n=31 *cxcr1*$^{-/-}$ (b,d) and n=21 wild type and n=21 *cxcr2*$^{-/-}$ (c,e). Unpaired t-test. Error bar represents S.E.M from individual embryos. p values are shown (p). Source data are provided as a Source Data file.

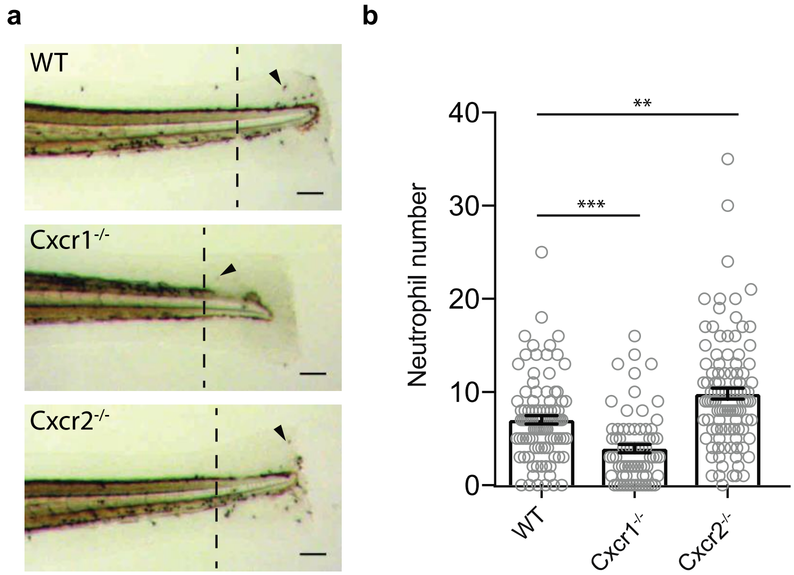

Figure 9. Cxcr1 and Cxcr2 inhibition facilitates and compromises resolution of inflammation respectively.

a) Bright field images of fixed, Sudan black-stained embryos at 24 hpw. Neutrophils appear as black dots of varying intensity. Arrows point to examples of cells. Neutrophil number within the proximal ventral, dorsal and tail fin were counted. For consistency, neutrophils were counted within a distance corresponding to about 300µm from the end of the notochord (dashed line). Scale bar = 100 µm. b) Quantification of neutrophil number. Error bars represent S.E.M. from individual embryos. Kruskal-Wallis test with Dunn’s multiple comparisons test. n=95 for WT (Tg(*mpx*:GFP)^{i114}), n=69 for Cxcr1$^{-/-}$ (*cxcr1*$^{-/-}$/Tg(*mpx*:GFP)^{i114}) and n=104 for Cxcr2$^{-/-}$ (*cxcr2*$^{-/-}$/Tg(*mpx*:GFP)^{i114}). Pooled from 4 experiments. Source data are provided as a Source Data file.

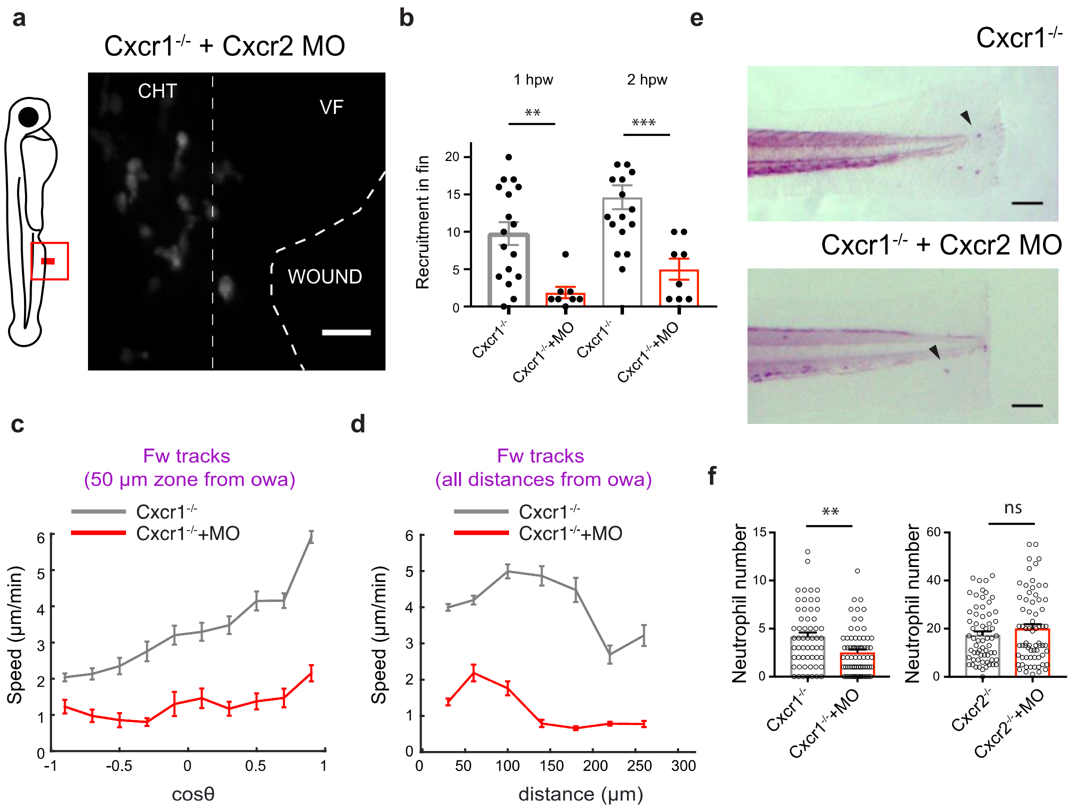

Figure 10. Cxcr2 knockdown in *cxcr1*$^{-/-}$ larvae inhibits chemotaxis to wounds.

a) Confocal projection images showing distribution of neutrophils at wounds at 1 hpw in *cxcr1*$^{-/-}$ (*cxcr1*$^{-/-}$/Tg(*mpx*:GFP)^{i114}) larvae injected with Cxcr2 morpholino. CHT = caudal hematopoietic tissue. VF = ventral fin. Cartoon of larva on the left indicates area imaged. Dashed lines show the outlines of VF and CHT. Scale bar = 25 µm. b) Neutrophil recruitment at 1 and 2 hpw, within a sample square field of 200µm length around the wound. n=17 (*cxcr1*$^{-/-}$) and n=8 (*cxcr1*$^{-/-}$ + MO) larvae, from 10 and 3 imaging sessions respectively. Two-tailed Mann-Whitney test. c) Neutrophil speed in relation to cosine of angle θ (cosθ). Average speeds per step per cosθ bin are shown. n=231-1436 steps per bin (*cxcr1*$^{-/-}$) and n=24-213 steps per bin (*cxcr1*$^{-/-}$ + MO) larvae. Track data within a zone of 0-50 µm from the owa are shown. d) Neutrophil speed in relation to distance from wound. Average speeds per cell per distance bin are shown. n=19-2922 steps per bin (*cxcr1*$^{-/-}$) and n=41-603 steps per bin (*cxcr1*$^{-/-}$ + MO) larvae.

c,d) Data are from 17 (*cxcr1*$^{-/-}$) and 8 (*cxcr1*$^{-/-}$ + MO) larvae from 10 and 3 imaging sessions respectively. e) Bright field images of fixed, sudan black-stained embryos at 4 hpw. Arrows point to examples of cells. Scale bar = 100 µm. f) Quantification of neutrophil number recruited in the tail fin. n=57 *cxcr1*$^{-/-}$ larvae, n=69 morpholino-injected *cxcr1*$^{-/-}$ larvae, n=64 *cxcr2*$^{-/-}$ larvae and n=68 morpholino-injected *cxcr2*$^{-/-}$ larvae. Two-tailed Mann-Whitney test. Error bars represent S.E.M. Source data are provided as a Source Data file.

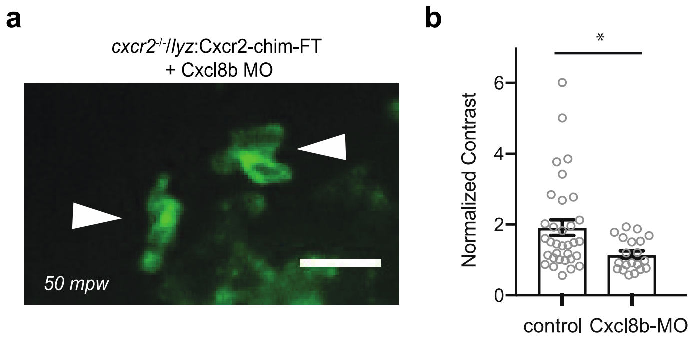

Figure. 11. Effect of Cxcl8b morpholino on Cxcr2-chim internalization in wounds.

a) Neutrophils in Tg(*lyz*:Cxcr2-chim-FT)/*cxcr2*$^{-/-}$ larvae treated with Cxcl8b morpholino. Time after wound is indicated. mpw = minutes post wound. Arrows point to two example neutrophils at the center of the wound, with representative distribution of the receptor. Representative image of non-injected control is shown in Fig. 4b. Scale bar = 15 µm. c) Quantification of contrast in Tg(*lyz*:Cxcr2-chim-FT)/*cxcr2*$^{-/-}$ neutrophils with or without morpholino treatment. n=33 cells from 8 larvae in 5 imaging sessions (control), n=20 cells from 4 independent larvae in 1 imaging session (Cxcl8b MO). Two-tailed Mann-Whitney test. Error bars represent S.E.M from individual cells. Source data are provided as a Source Data file.

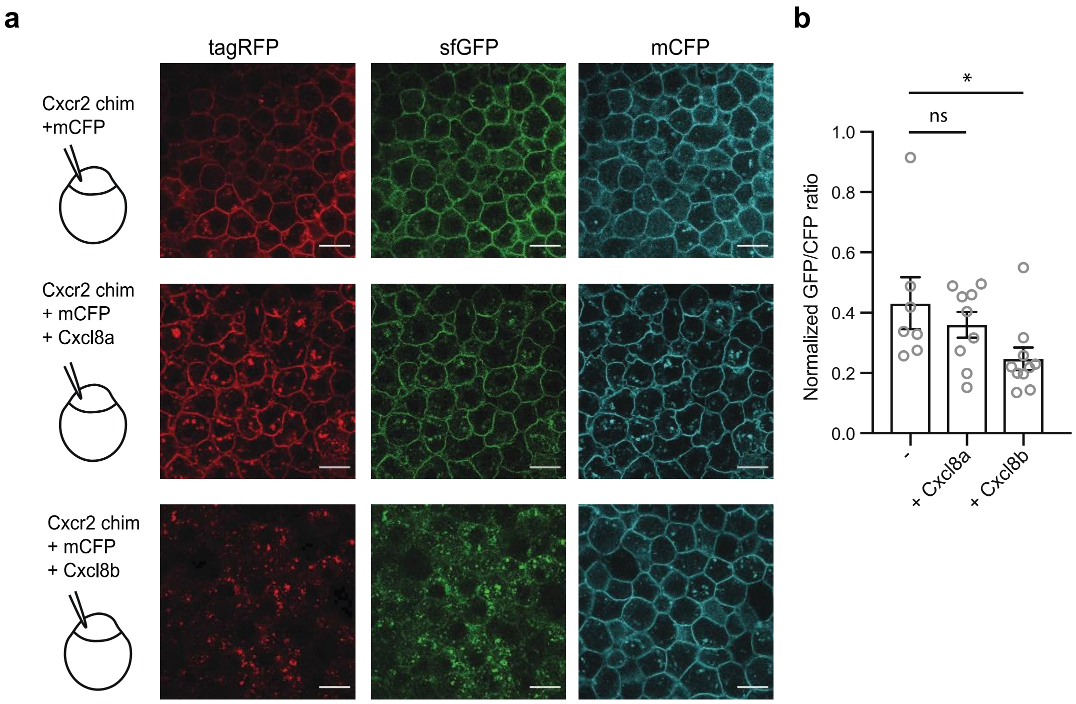

Figure 12. Cxcr2-chim internalizes in response to Cxcl8b and not Cxcl8a.

a) 100pg of Cxcr2-chim-FT mRNA was injected with or without 150pg of Cxcl8a or Cxcl8b mRNA in one-cell stage embryos. Laser-scanning confocal slices of gastrulating embryos show expression and distribution of Cxcr2-chim-FT. Green and red receptors are shown in separate channels. Control membrane marker (mCFP) is shown in the cyan channel. Scale bar = 20 µm. b) Quantification of Cxcr2-chim-FT membrane expression as a function of mCFP, normalized as in Supplementary figures 3 and 7. n=7 embryos for Cxcr2-chim alone from 4 imaging sessions, n=9 Cxcr2-chim + Cxcl8a embryos from 3 imaging sessions and n=10 Cxcr2-chim + Cxcl8b embryos from 2 imaging sessions. Kruskal-Wallis test with Dunn's multiple comparison test. Error bars represent S.E.M from individual embryos. Source data are provided as a Source Data file.

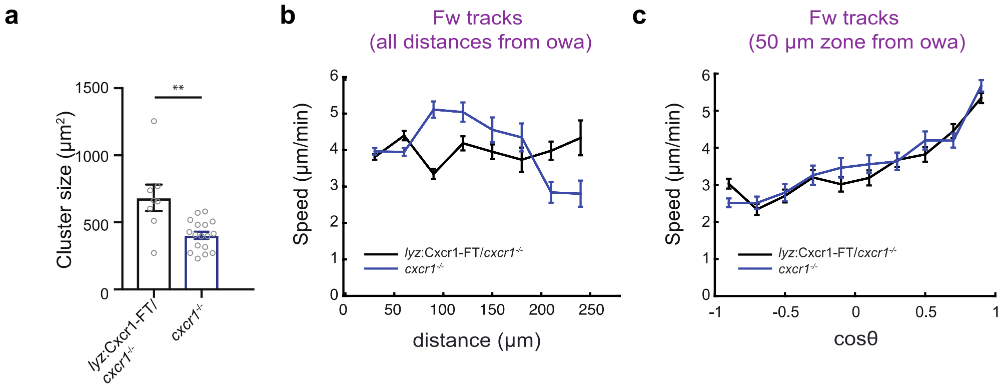

Figure 13. Neutrophil expression of Cxcr1-FT rescues clustering defect.

a) Average neutrophil cluster size per larva throughout the first 2 hpw. n=17 *cxcr1*$^{-/-}$ and n=8 Tg(*lyz:*Cxcr1-FT*)/cxcr1*$^{-/-}$ larvae. Two-tailed Mann-Whitney test. b) Neutrophil speed in relation to distance from wound. Average speeds per cell per distance bin are shown. n=144-3008 steps per bin for *cxcr1*$^{-/-}$ and n=59-3396 steps per bin for Tg(*lyz:*Cxcr1-FT*)/cxcr1*$^{-/-}$ larvae. c) Neutrophil speed in relation to cosine of angle θ. Average speeds per cell per cosθ bin are shown. n=231-1436 steps per bin for *cxcr1*$^{-/-}$ and n=206-1409 steps per bin for Tg(*lyz:*Cxcr1-FT*)/cxcr1*$^{-/-}$ larvae. For all panels, data are from 17 *cxcr1*$^{-/-}$ larvae and 8 Tg(*lyz:*Cxcr1-FT*)/cxcr1*$^{-/-}$ larvae from 10 and 4 imaging sessions respectively. Error bars represent S.E.M from individual embryos (a) or cell steps (b,c). Source data are provided as a Source Data file.

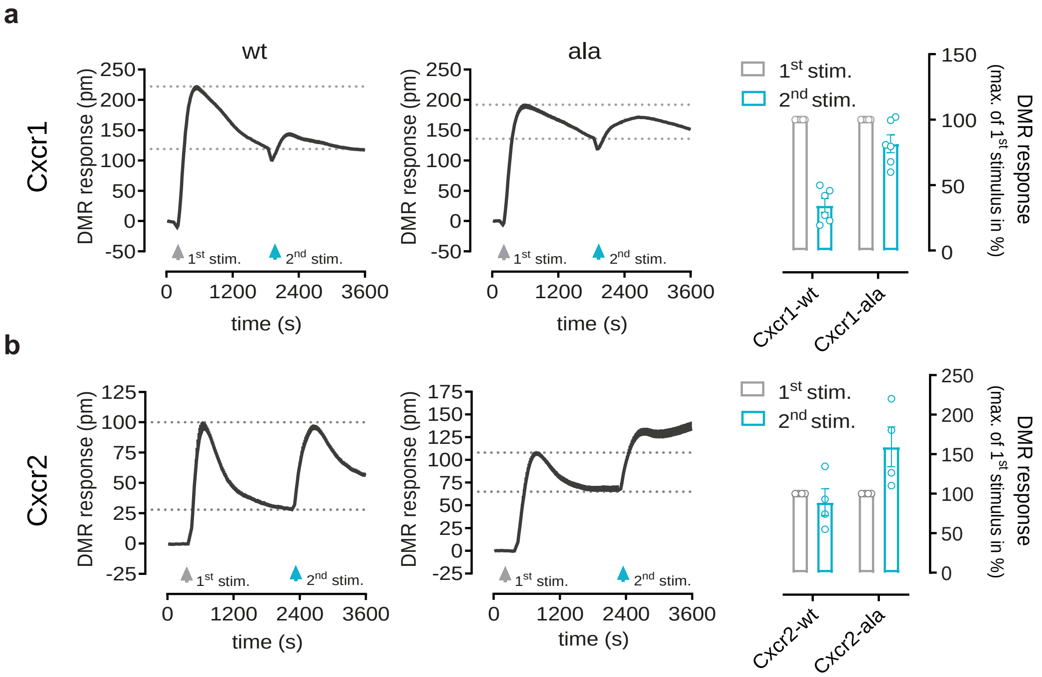

Figure 14. Cxcr1 and Cxcr2 show distinct signaling profiles after repeated exposure to their cognate ligands.

Real-time DMR recordings of whole cell activity induced by repeated application of saturating concentrations of Cxcl8a (80 nM), for Cxcr1 stimulation, or Cxcl8b (supernatant, 1:4 dilution), for Cxcr2 stimulation, in HEK293 cells stably expressing the indicated chemokine receptor constructs. Arrows indicate application of the first and second ligand stimulus. Blue bars depict the fractional response of the second stimulus relative to the first response for each receptor. Fractional recovery of receptor responses were calculated based on the maximum amplitude of the first stimulus (upper dotted line, 100%) and the minimum amplitude (lower dotted line, 0%) prior to addition of the second stimulus. Real-time DMR recordings are representative for each condition and depict the mean + S.E.M. of a technical triplicate. Error bars in bar graphs represent S.E.M. of 6 (Cxcr1) or 4 (Cxcr2) independent biological replicates. Source data are provided as a Source Data file.

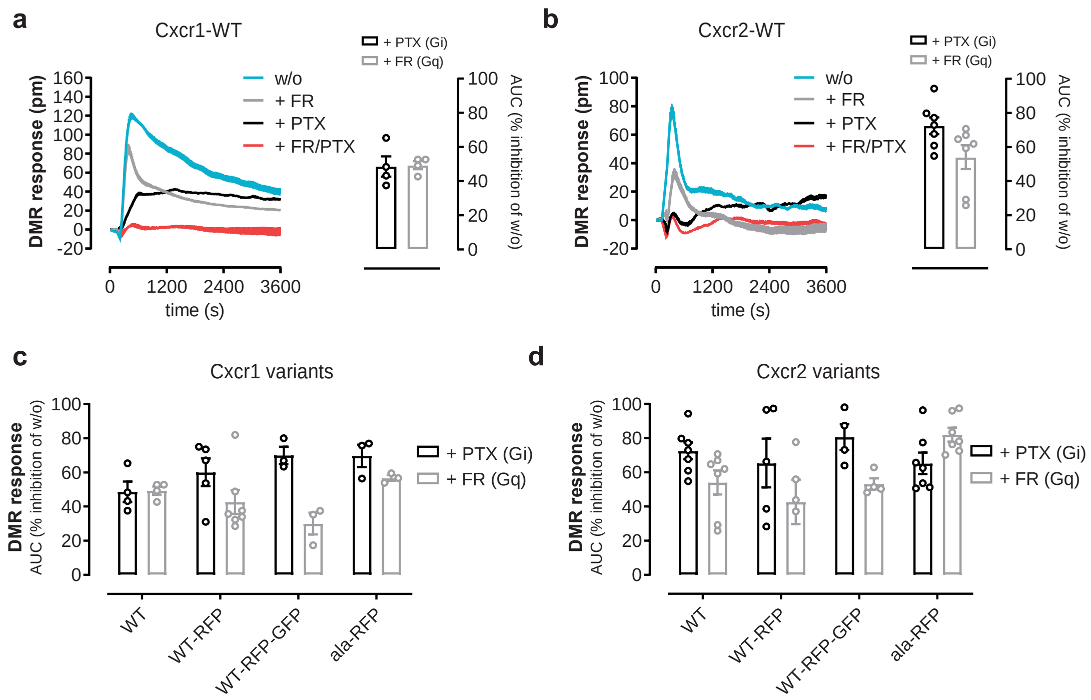

Figure 15. Cxcr1 and Cxcr2 transduce cellular signals by activating heterotrimeric Gi and Gq proteins.

(a,b) DMR recordings of Cxcl8a-activated Cxcr1 and of Cxcl8b-activated Cxcr2 in HEK293 cells pretreated or not with Gi inhibitor pertussis toxin (PTX), Gq inhibitor FR900359 (FR) or a combination of both. Shown are representative real-time DMR traces along with bar diagrams depicting Gi and Gq contribution for each receptor (i.e. inhibition by PTX and FR respectively). Fractional Gi and Gq engagement (AUC, area under the curve) was calculated as percent inhibition from normalized whole cell responses in the absence of inhibitor treatment (without, w/o). (c,d) G protein recognition of untagged Cxcr1 and Cxcr2 wildtype receptors and their fluorescently labeled and mutant variants. Real-time DMR recordings are representative for each condition and depict the mean + S.E.M. of a technical triplicate. Error bars in bar graphs indicate S.E.M. from 3-7 independent biological replicates. Source Data are provided as a Source Data file.
