# Supplementary material for: Chemokine receptor trafficking coordinates neutrophil clustering and dispersal at wounds in zebrafish
Source: Nat Commun. 2019 Nov 14;10:5166. doi: 10.1038/s41467-019-13107-3 (PMC6856356; doi:10.1038/s41467-019-13107-3)
Supplement: Supplementary file 3 — Description of Additional Supplementary Files [file 41467_2019_13107_MOESM3_ESM.pdf]

## Supplementary Movie Legends

**Supplementary Movie 1.** Transgenic neutrophils in the head of a Tg(*lyz*:Cxcr1-FT) (left) and Tg(*lyz*:Cxcr2-FT) (right) larva at 3 dpf. sfGFP(green), tagRFP (magenta). Frame interval is 30 sec and frame rate is 5 fps. Scale bar = 20 µm.

**Supplementary Movie 2.** Neutrophils in Tg(*lyz*:Cxcr1-FT) (left) and Tg(*lyz*:Cxcr2-FT) (right) transgenic larvae responding to fin wounds. Movie starts within 10 mpw and lasts 60 min. sfGFP (green), tagRFP (magenta). Frame interval is 30 sec and frame rate is 10 fps. CHT = caudal hematopoietic tissue. VF = ventral fin. Scale bar = 25 µm.

**Supplementary Movie 3.** Additional examples of neutrophils from a wounded Tg(*lyz*:Cxcr1-FT) transgenic larva (different larva to that shown in Video 2), acquired at higher resolution, showing receptor internalization (sfGFP channel shown in green) upon mobilization in the CHT or upon entry and chemotaxis in the ventral fin. Frame interval is 30 sec and frame rate is 2 fps. Scale bar = 10 µm.

**Supplementary Movie 4.** Zoomed view of neutrophils from Video 2, focused at the wound, showing receptor internalization (sfGFP channel shown in green) in Tg(*lyz*:Cxcr1-FT) (left) and Tg(*lyz*:Cxcr2-FT) (right) transgenic larvae responding to fin wounds. Movie starts within 2 mpw and lasts 60 min. Frame interval is 30 sec and frame rate is 10 fps. Scale bar = 25 µm.

**Supplementary Movie 5**. Neutrophils in Tg(*lyz*:Cxcr1-FT) transgenic larvae treated with *cxcl8a* morpholino (MO) (left) and *cxcl8b* MO (right) responding to fin wounds (sfGFP channel shown in green). Area shown has the same size as in Video 4 and is focused at the wound. Movie starts at 85 mpw in left movie and 37 mpw in right movie. Frame interval is 120 sec, frame rate is 5 fps and movie lasts 7 min. Scale bar = 25 µm.

**Supplementary Movie 6.** Neutrophils (green) in wild type Tg(*mpx:*GFP)$^{i114}$ (top panels) or Tg(*mpx*:GFP)$^{i114}$/cxcr1$^{-/-}$ (bottom panels, cxcr1 KO) in the presence of transplanted Cxcl8-mCherry-secreting cells (magenta). Left movies show merged channels and right movies show only magenta fluorescence to facilitate visualization of internalized Cxcl8-mCherry. Internalization can be observed as a punctate pattern of magenta vesicles inside neutrophils. The movie lasts for 30 min. Frame interval is 30 sec and frame rate is 5 fps. Scale bar = 25 µm.

**Supplementary Movie 7.** Neutrophils in Tg(*lyz*:Cxcr1-FT) transgenic larvae responding to wounds (sfGFP channel shown in green). LTB4 was added in the bath of the larva and neutrophils entered the ventral fin within 5 min (PRE-WOUND). Larvae were wounded 30 minutes after addition of LTB4 and Cxcr1-FT internalization in interstitial neutrophils was monitored and quantified (POST-WOUND; imaging starts within 15 mpw and lasts for 1 h). Frame interval is 30 sec and frame rate is 10 fps. CHT = caudal hematopoietic tissue. VF = ventral fin. Scale bar = 25 µm.

**Supplementary Movie 8.** Neutrophils responding to a ventral fin wound in wild type Tg(*mpx*:GFP)$^{i114}$ transgenic larvae, Tg(*mpx*:GFP)$^{i114}$/*cxcr1*$^{-/-}$ transgenic larvae (Cxcr1 KO), Tg(*mpx*:GFP)$^{i114}$/*cxcr2*$^{-/-}$ transgenic larvae (Cxcr2 KO) or Tg(*mpx*:GFP)$^{i114}$/cxcr1$^{-/-}$ transgenic larvae injected with *cxcr2* morpholino (Cxcr1 KO+Cxcr2 MO). GFP channel is shown in gray. Movie starts within 15 mpw and lasts about 2 h. Frame interval is 30 sec and frame rate is 20 fps. CHT = caudal hematopoietic tissue. VF = ventral fin. Scale bar = 50 µm.

**Supplementary Movie 9.** Receptor distribution (sfGFP channel shown in green) in neutrophils (sfGFP shown) from representative Tg(*lyz*:Cxcr1-WT-FT)/*cxcr1*$^{-/-}$, Tg(*lyz*:Cxcr1-ala-FT)/*cxcr1*$^{-}$

$^{/-}$ and Tg(*lyz*:Cxcr1-chim-FT)/*cxcr1*$^{-/-}$ larvae. Movie begins approximately 2 hpw. Frame interval is 30 sec and frame rate is 5 fps. Timestamp is shown in minutes. W = wound. Scale bar = 32 µm.

**Supplementary Movie 10**. Receptor distribution (sfGFP channel shown in green) in neutrophils from representative Tg(*lyz*:Cxcr2-WT-FT)/*cxcr2*$^{-/-}$, Tg(*lyz*:Cxcr2-ala-FT)/*cxcr2*$^{-/-}$ and Tg(*lyz*:Cxcr2-chim-FT)/*cxcr2*$^{-/-}$ larvae. Movie begins at different time points (minutes) post-wound as indicated at the top left. Tg(*lyz*:Cxcr2-chim-FT)/*cxcr2*$^{-/-}$ larvae are shown from the beginning of recruitment as the receptor distribution changes over time. Frame interval is 30 sec and frame rate is 5 fps. Timestamp is shown in minutes. W = wound. Scale bar = 25 µm.

**Supplementary Movie 11**. Receptor distribution (sfGFP channel shown in green) in neutrophils from a representative Tg(*lyz*:Cxcr2-FT-chim)/*cxcr2*$^{-/-}$ larva injected with Cxcl8b MO. Movie begins 40 mpw as indicated at the top left. Arrows point to two exemplary cells showing receptor internalization but also residual membrane expression. These cells disperse from the wound at the end the movie. Timepoint after wound is shown in minutes. Frame interval is 60 sec and frame rate is 5 fps. mpw = minutes post wound. W = wound. Scale bar = 25 µm.

**Supplementary Movie 12.** Neutrophil behavior and distribution in representative Tg(*mpx*:GFP)$^{i114}$/Tg(*lyz*:Cxcr2-FT-WT)/*cxcr2*$^{-/-}$, Tg(*mpx*:GFP)$^{i114}$/Tg(*lyz*:Cxcr2-FT-ala)/*cxcr2*$^{-/-}$ and Tg(*mpx*:GFP)$^{i114}$/Tg(*lyz*:Cxcr2-FT-chim)/*cxcr2*$^{-/-}$ larvae (sfGFP/GFP channel shown in green). Movie begins 25 mpw, 15 mpw and 15 mpw respectively. Frame interval is 30 sec and frame rate is 20 fps. W = wound. Scale bar = 25 µm.

**Supplementary Movie 13.** Zoomed out version of Video 9 showing neutrophil behavior and distribution in representative Tg(*lyz*:Cxcr1-FT-WT)/*cxcr1*$^{-/-}$, Tg(*lyz*:Cxcr1-FT-ala)/*cxcr1*$^{-/-}$ and Tg(*lyz*:Cxcr1-FT-chim)/*cxcr1*$^{-/-}$ larvae (sfGFP channel shown in green). Movie begins approximately 2 hpw. Frame interval is 30 sec and frame rate is 20 fps. W = wound. Scale bar = 32 µm.
